# Circulating Proteomics and Risk of Atrial Fibrillation: A Systematic Review of Cohort Studies

**DOI:** 10.1111/jcmm.70760

**Published:** 2025-08-05

**Authors:** Luxiang Shang, Weilin Wang, Yiying Liu, Baopeng Tang, Yinglong Hou

**Affiliations:** ^1^ Department of Cardiology The First Affiliated Hospital of Shandong First Medical University and Shandong Provincial Qianfoshan Hospital Jinan China; ^2^ Medical Science and Technology Innovation Center Shandong First Medical University and Shandong Academy of Medical Sciences Jinan China; ^3^ National Laboratory of Biomacromolecules, Institute of Biophysics Chinese Academy of Sciences Beijing China; ^4^ Department of Pacing and Electrophysiology, Xinjiang Key Laboratory of Cardiac Electrophysiology and Remodeling The First Affiliated Hospital of Xinjiang Medical University Urumqi China

**Keywords:** atrial fibrillation, biomarkers, proteomics, risk stratification, systematic review

## Abstract

Atrial fibrillation (AF) is a common arrhythmia associated with significant morbidity and adverse outcomes. High‐throughput proteomics offers a promising approach for identifying circulating biomarkers to improve AF risk stratification. This systematic review evaluated cohort studies published from 2010 to 2024 that employed proteomic approaches to investigate associations between circulating proteins and AF incidence. Studies were screened using predefined criteria, and proteins significantly associated with AF in fully adjusted models were extracted. Bioinformatics analyses, including pathway enrichment and protein–protein interaction (PPI) network mapping, were conducted to characterise the implicated biological processes. Proteins reproducibly associated with AF across studies were further evaluated through drug target annotation and assessed for potential causal relationships using Mendelian randomisation (MR). In total, 111 proteins were identified across nine cohorts, with significant enrichment in pathways related to extracellular matrix organisation and cell adhesion. PPI analysis identified interleukin‐6, matrix metalloproteinase‐2 and C‐reactive protein as central network hubs. Thirteen proteins were reproducible across at least two studies, with NT‐proBNP consistently reported in eight cohorts, supporting its role as a robust and reliable biomarker of AF risk. Drug target analysis showed that four proteins—ANGPT‐2, adrenomedullin, GDF15 and PLAUR—are currently under clinical investigation. MR analysis did not confirm causal associations between these proteins and AF, suggesting they may reflect disease‐related processes rather than directly drive AF onset. This review highlights reproducible proteomic biomarkers and their functional networks in AF, providing a foundation for future studies focused on biomarker‐guided prevention and therapeutic development.

**Trial Registration:** PROSPERO number: CRD420251063038

## 1 Introduction

Atrial fibrillation (AF) is the most common arrhythmia, with a lifetime risk of approximately one‐fourth to one‐third [1]. It is a major public health concern, contributing to significant morbidity and mortality, including complications such as stroke, heart failure, and cognitive decline [2]. As the global population continues to age, the incidence and prevalence of AF are expected to rise, further emphasising the urgent need for effective strategies to identify high‐risk individuals [3]. Risk prediction can enable individualised assessment, timely identification of high‐risk individuals and targeted intervention.

To date, over 20 community‐based AF prediction models have been developed, incorporating conventional clinical variables such as age, hypertension, diabetes and body mass index [4]. However, none of these models have shown superior predictive performance, and their accuracy remains suboptimal [5]. Notably, these models often neglect molecular biomarkers, which could significantly enhance predictive capabilities. Identifying novel biomarkers and integrating them into risk models is therefore a crucial step towards improving AF prevention and management.

Proteomics, a high‐throughput technology for large‐scale protein quantification, has become an invaluable tool in cardiovascular biomarker research [6, 7]. Advanced platforms such as Olink, which uses Proximity Extension Assay (PEA) technology and SomaScan, employing SOMAmer aptamers, allow for the highly sensitive detection of thousands of proteins from minimal sample volumes [8]. These technologies enable precise biomarker discovery, particularly for complex conditions like AF, with SomaScan quantifying over 11,000 proteins and Olink over 5000 proteins [8].

Recent studies have leveraged proteomics to identify protein biomarkers associated with AF [9, 10, 11, 12, 13, 14, 15, 16, 17]. While these findings offer valuable insights, inconsistencies across studies remain due to differences in design, populations and proteomic platforms. This study systematically reviews cohort studies employing proteomics in AF research, aiming to identify reproducible protein biomarkers. By synthesising these findings, it seeks to improve AF risk stratification and provide new insights into its molecular underpinnings.

## 2 Methods

### 2.1 Study Design

This study employed a comprehensive, multi‐layered approach to evaluate proteins associated with AF incidence as reported in cohort studies. First, a systematic review identified relevant studies using high‐throughput proteomics to investigate circulating proteins linked to AF. Next, bioinformatics analyses, including Gene Ontology (GO), Kyoto Encyclopedia of Genes and Genomes (KEGG) and protein–protein interaction (PPI) network analyses, were conducted to explore the biological functions and pathways associated with these proteins. Proteins consistently identified across studies were labelled as reproducible proteins, reflecting robust candidates for further analysis. Reproducible proteins were further evaluated using Mendelian randomisation (MR) to assess causal relationships with AF and atrial remodelling. To strengthen the causal evidence, transcriptome‐wide association studies (TWAS) and colocalisation analyses were also performed. Finally, drug target analysis was conducted to evaluate the therapeutic potential of these proteins. This structured design provided a comprehensive framework for identifying robust biomarkers and exploring their biological and clinical relevance.

This study adhered to the Preferred Reporting Items for Systematic Reviews and Meta‐Analyses (PRISMA) guidelines [18]. The study protocol has been registered in PROSPERO (CRD420251063038). Since this study is a systematic review of published literature and did not involve any patient‐level data, it was exempt from informed consent and ethics review. The genome‐wide association studies (GWAS) included in the MR analyses had received ethical approval from the relevant ethics committees, and written informed consent was obtained from all participants involved in those studies.

### 2.2 Literature Search

Two independent investigators (L.S. and W.W.) conducted a systematic literature search in PubMed, Embase and Web of Science databases, covering studies published between 2010 and November 4, 2024. Discrepancies in study inclusion were resolved by a senior cardiologist (B.T.). The search strategy incorporated key terms such as ‘atrial fibrillation’, ‘proteomics’ and ‘cohort study’. Detailed search strategies for each database are provided in Table S1.

### 2.3 Study Selection

Studies were selected based on the PICOS framework [19] as follows: (1) *Population*: general population, excluding specific patient groups such as those with heart failure; (2) *Intervention*: high‐throughput proteomic analysis of blood samples, including sequencing methods targeting specific proteins; (3) *Outcome*: incidence of AF, with no restrictions on diagnosis method or AF subtype; (4) *Study design*: cohort studies employing Cox proportional hazards models to evaluate protein‐AF associations; (5) *Language*: articles published in English. Exclusion criteria included case reports, reviews, editorials, letters, animal studies and non‐full‐length publications.

### 2.4 Data Collection

Two authors independently extracted relevant study data, including first author, publication date, study design, study location, sample size, baseline age, sex, follow‐up duration, number of confirmed AF cases and key conclusions. Discrepancies were resolved by consensus involving a third investigator. A pre‐designed electronic form was used to standardise data extraction.

Study quality was assessed using a modified Newcastle‐Ottawa Scale (NOS) adapted for proteomics research [20]. Traditional NOS domains were retained, while an additional domain titled Proteomics was introduced to better capture the methodological rigour of high‐throughput protein biomarker studies [21]. The Selection domain evaluated case definition, representativeness of cases and controls and control selection. The Comparability domain assessed whether studies adequately adjusted for key confounders such as age and sex. In the Proteomics domain, we included three specific items relevant to the design and interpretability of proteomic studies: (1) Methodology, reporting, preparation and analysis—whether the study clearly described the proteomic platform, sample handling and analytic pipeline. (2) Human database identification—whether protein identifiers (e.g., UniProt IDs) were reported to facilitate cross‐study comparison. (3) Quantification—whether absolute quantification (e.g., in ng/mL) was provided. Each item was awarded one point if the criterion was met, resulting in a maximum total score of 9.

### 2.5 Identification of Proteins Associated With AF Incidence

We extracted proteins associated with AF incidence from multivariable regression models that adjusted for confounders such as sex, age and clinical risk factors, as these proteins reflect independent associations with AF. Protein information, including UniProt IDs [22], protein names, gene names and PANTHER protein classes [23], was manually curated from published studies. Additional details were verified using manufacturer databases, including SomaLogic (www.somalogic.com) and Olink (www.olinkexplore.com). Each protein was checked individually, emphasising manual verification. Proteins identified in two or more studies were designated as reproducible proteins, representing robust candidates for further analysis.

### 2.6 Bioinformatics Analysis

To investigate the biological functions of AF‐associated proteins, we conducted comprehensive bioinformatics analyses using UniProt gene IDs and multiple databases. Functional and pathway enrichment analyses were performed with GO, KEGG, Reactome [24] and WikiPathways [25] to identify significantly enriched biological processes, molecular functions, cellular components and signalling pathways. Protein–protein interactions (PPI) among AF‐related proteins were assessed using the STRING database [26] to construct PPI networks and identify core proteins. For reproducible proteins, interaction networks were constructed via the BioGRID database [27] and analysed using network topology analysis (NTA) on the WebGestalt platform [28]. To ensure comprehensive coverage, the expanded network incorporated the top 50 neighbouring nodes for each protein, with significance determined by the top 10 functional nodes.

### 2.7 Drug Target Analysis

Drug target analysis was conducted for reproducible proteins using databases such as the Therapeutic Target Database (TTD) [29], Drug–Gene Interaction Database [30], DrugBank [31] and The Open Targets Platform [32]. This analysis aimed to identify whether these proteins are targeted by approved or investigational small molecules or biologics, providing insights into their therapeutic potential.

### 2.8 Study Design and Data Sources for MR Analysis

Causal relationships between reproducible proteins, AF, and atrial remodelling were assessed using a two‐sample MR approach. Genetic instruments for circulating proteins were derived from cis‐pQTL data obtained from 35,559 individuals of Icelandic descent, covering 4907 proteins [33]. GWAS summary statistics for AF were sourced from the largest available international consortium study [34], which included 60,620 AF cases and 970,216 controls, as well as the FinnGen study, which provided additional population‐based data [35]. Additionally, GWAS data for four atrial structural and functional traits (left atrial minimum volume, maximum volume, stroke volume and emptying fraction) were obtained from the UK Biobank [36].

To further validate the associations, transcriptome‐wide association studies (TWAS) were conducted using eQTLGen [37], a dataset including 31,684 blood samples and GTEx (v8) project [38], which contains cis‐eQTL data for multiple tissues, including blood, atrial and ventricular tissues. The sources and detailed information of the GWAS datasets used are provided in Table S2. Colocalisation analyses were performed to determine whether protein‐AF associations were influenced by shared causal variants, using a Bayesian framework to calculate posterior probabilities.

### 2.9 Statistical Analysis of MR

Statistical analyses were performed using R software (version 4.4.1) and relevant packages, including TwoSampleMR, MRPRESSO and coloc, as well as the smr‐1.3.1‐win software. Statistical significance was set at a Bonferroni‐corrected threshold, and all statistical tests for the MR analysis were two‐sided. For proteins with multiple cis‐pQTLs, the inverse variance weighted method was used to estimate causal effects, while the Wald ratio method was applied for proteins with a single instrumental variable [39, 40]. Odds ratios (OR) and 95% confidence intervals (CI) were calculated for the risk of AF per 1‐SD increase in protein expression. For TWAS, the SMR approach was used to evaluate associations between gene expression and AF risk, selecting the most significant cis‐eQTL SNP as the instrumental variable [41].

Colocalization analysis was used to determine whether the association between proteins and AF was driven by the same causal variants. This analysis was conducted using a Bayesian model to evaluate the posterior probabilities for five hypotheses: (1) no association; (2) association with only one trait; (3) association with the other trait; (4) association with both traits driven by distinct variants; and (5) association with both traits driven by shared causal variants. Posterior probabilities > 0.8 were interpreted as strong evidence for colocalization, while values > 0.5 indicated moderate evidence [42].

## 3 Results

### 3.1 Characteristics and General Information of the Included Studies

**FIGURE 1:**
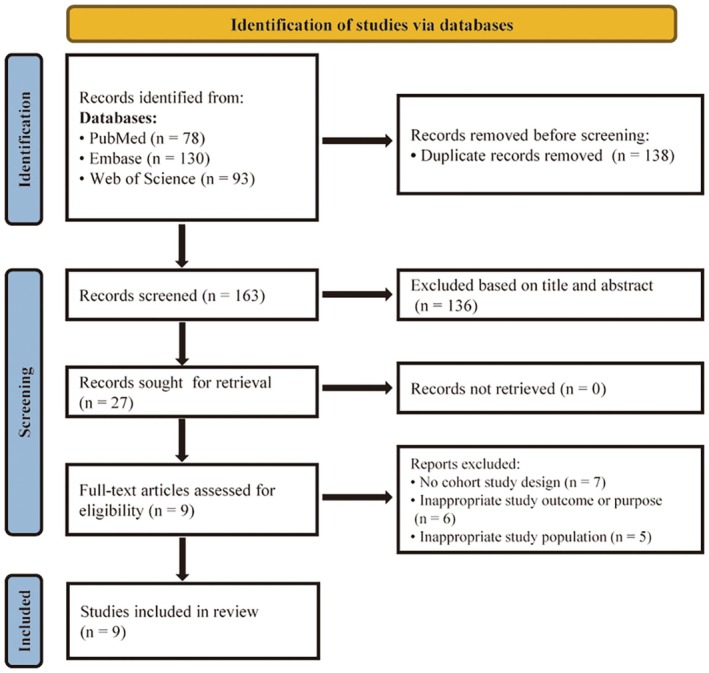
PRISMA diagram of the publication search and identification.

After removing duplicates from the database search, 163 citations were identified (Figure 1). Following title and abstract screening, 27 articles were selected for full‐text review, of which 18 were excluded based on predefined criteria. Ultimately, 9 articles met the inclusion criteria and were included in this study [9, 10, 11, 12, 13, 14, 15, 16, 17]. These studies, published between 2017 and 2024, were based on cohorts from five countries: five in the United States, three in Sweden, and one each in Finland, Iceland, and the United Kingdom. Sample sizes varied considerably, ranging from 725 to 38,784 participants. The most commonly used proteomics platforms were Olink and SomaScan. Detailed characteristics of the included cohort studies are presented in Table 1.

**TABLE 1:** Characteristics and main findings of included studies.

First author, year	Study design	Registry/study name	Study location	Enrollment	Sample size	Age (years)	Female (%)	Follow‐up time (years)	Incident AF cases	Biological fluids used	Proteomic profiling platform/assay	Major findings
Lind et al., 2017	Community‐based cohort	Discovery cohort: Prospective Investigation of the Vasculature in Uppsala Seniors (PIVUS)	Sweden	2001–2004	978	70.1 ± 0.1	51	10.0	148	Plasma	Olink Cardiovascular panel (92 proteins)	13 proteins were related to incident AF in PIVUS and 5 proteins were replicated in ULSAM 7 proteins were related to incident AF for a meta‐analysis based on individual data
	Community‐based cohort	Replication cohort: Uppsala Longitudinal Study of Adult Men (ULSAM)	Sweden	1997–2001	725	77.5 ± 0.7	0	7.9	123			
Ko et al., 2019	Community‐based cohort	Framingham Heart Study (FHS)	USA	1991–1995	1885	55 ± 10	54	18.3	349	Plasma	SomaScan (1373 proteins)	8 proteins were associated with risk of incident AF after adjustment for age and sex 2 proteins (ADAMTS13 and NT‐proBNP) were associated with AF after further adjustment for clinical variables
Staerk et al., 2020	Community‐based cohort	Framingham Heart Study (FHS)	USA	1998–2001, 2002–2005	3378	61.5 ± 8.4	54.4	12.3 ± 3.8	401	Plasma	Luminex xMAP (85 proteins)	3 proteins (IGF1, IGFBP1, and NT‐proBNP) were associated with the risk of incident AF
Molvin et al., 2020	Community‐based cohort	Malmö Preventive Project (MPP)	Sweden	2002–2006	1694	69.5	29.3	9.7 ± 3.1	278	Plasma	Olink CVD III panel (92 proteins)	5 proteins were associated with incident AF in fully‐adjusted model
Norby et al., 2021	Community‐based cohort	Atherosclerosis Risk in Communities (ARIC) Study	USA	2011–2013	4668	75 ± 5	59	5.7 ± 1.7	585	Plasma	SomaScan (4877 proteins)	37 proteins were associated with risk of incident AF after adjustment for variables of CHARGE‐AF risk score 17 proteins were associated with AF after further adjustment for eGFR and medication use
Chen et al., 2022	Community‐based cohort	Atherosclerosis Risk in Communities (ARIC) Study	USA	1993–1995	10,234	60.0 ± 5.7	54.9	20.6	2217	Plasma	SomaScan	GDF‐15 was associated with incident AF after adjusting for clinical confounders and 2 cardiac biomarkers (cardiac troponin T and natriuretic peptide)
Börschel et al., 2023	Population‐based cohort	FINRISK cohort	Finland	1997, 2002	10,744	50.9	51.3	20.8	1246	NA	Bio‐Plex Pro Human Cytokine 27‐plex Assay and 21‐plex Assay	4 proteins were associated with risk of incident AF after adjustment for age and sex NT‐proBNP was associated with AF after further adjustment for clinical variables
Jonmundsson et al., 2023	Population‐based cohort	Discovery cohort: Age, Gene/Environment Susceptibility‐Reykjavik study (AGES‐RS)	Iceland	2002–2006	4765	76 (72–80)	59.3	10.5 (6.2–14.1)	1172	Serum	SomaScan (4137 proteins)	76 proteins were significantly associated with incident AF in AGES‐RS 29 proteins were replicated successfully in CHS cohort 9 proteins were identified to have a causal relationship with AF in the Mendelian randomization analysis
	Population‐based cohort	Replication cohort: Cardiovascular Health Study (CHS)	USA	NA	3305	72 ± 5	62	11 ± 7	1338	NA	SomaScan (7k and 5k proteins)	
Peng et al., 2024	Population‐based cohort	UK Biobank Pharma Proteomics Project (UKB‐PPP)	UK	NA	38,784	58 (50–63)	54.1	14.5 (discovery cohort)	2345	Plasma	Olink Explore 3072 panel (2923 proteins)	21 over‐lapping proteins were associated with incident AF in UKB‐PPP cohort 2 proteins (COL4A1 and RET) had a causal relationship with AF in the Mendelian randomization analysis

Abbreviations: ADAMTS13, a disintegrin and metalloproteinase with thrombospondin motifs 13; AF, atrial fibrillation; COL4A1, collagen IV α‐1; GDF‐15, growth differentiation factor 15; IGF1, insulin‐like growth factor I; IGFBP1, insulin‐like growth factor‐binding protein 1; NA, not available; NT‐proBNP, N‐terminal pro‐B‐type natriuretic peptide; RET, proto‐oncogene tyrosine‐protein kinase receptor Ret.

Among the nine included studies, two were based on the ARIC cohort [13, 14]. Despite sharing a cohort source, these studies differed substantially in design, baseline visit and proteomic scope. One utilised high‐throughput profiling of thousands of proteins measured at Visit 5 (2011–2013), while the other focused on a single‐protein measurement from Visit 3 (1993–1995). Due to these methodological differences and non‐reproducible biomarker assessments, both studies were considered eligible and included in the review.

### 3.2 Quality Assessment of Included Studies

All included studies were evaluated as having a low risk of bias (Table 2). The most common limitation was the lack of absolute quantification, as targeted proteomics platforms like Olink and SomaScan typically provide relative rather than absolute protein expression levels. Another notable issue was that, while all studies reported full protein names and abbreviations for AF‐associated proteins, only one provided detailed identifiers such as UniProt IDs.

**TABLE 2:** Studies' quality using the modified Newcastle–Ottawa scale.

Source	Selection				Comparability		Proteomics			Total score
	Case definition	Cases representativeness	Controls selection	Controls representativeness	Age‐controlled	Sex‐controlled	Methodology, reporting, preparation, analysis	Human database identification	Quantification	
Lind et al., 2017	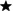	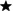	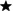	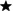	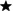	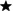	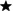			7
Ko et al., 2019	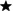	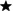	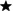	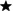	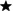	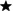	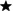			7
Staerk et al., 2020	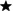	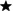	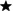	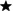	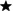	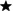	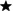			7
Molvin et al., 2020	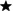	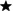	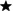	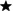	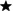	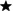	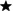			7
Norby et al., 2021	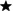	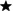	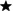	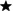	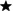	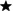	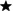			7
Chen et al., 2022	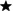	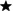	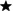	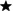	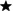	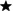	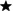			7
Börschel et al., 2023	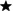	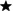	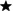	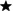	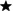	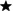	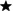			7
Jonmundsson et al., 2023	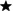	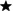	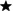	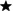	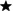	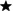	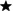			7
Peng et al., 2024	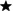	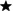	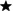	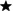	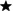	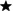	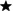	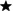		8

*Note:* The 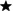 symbols indicate the results of the quality assessment using the Newcastle‐Ottawa Scale (NOS). Each star represents that the study fulfills the corresponding quality criterion in the NOS. The total number of stars reflects the overall methodological quality of each included study.

### 3.3 AF‐Associated Proteins and Bioinformatics Analysis

**FIGURE 2:**
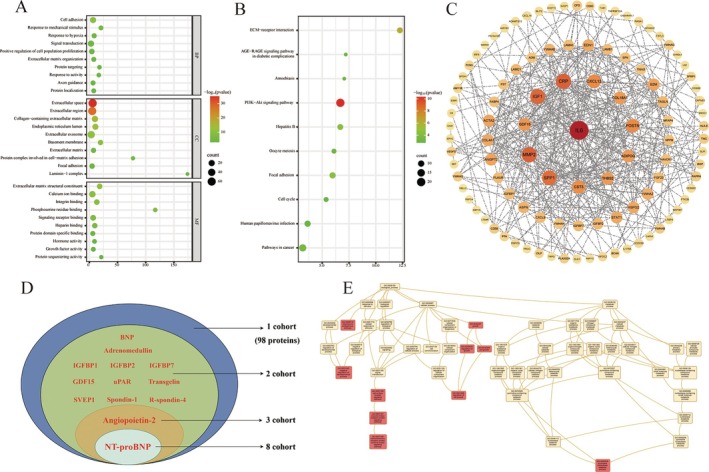
Proteomics associated with the incidence of AF in cohort studies included in this systematic review. (A) Functional analysis of the biomarker pool showing the top 10 Gene Ontology terms related to biological processes, cellular components, and molecular functions. (B) The top 10 pathways identified through KEGG enrichment analysis. (C) Protein–protein interaction network generated using the STRING database. (D) Reproducibility of 111 protein biomarkers across nine independent studies. Proteins in red are positively associated with AF incidence. (E) Network topology enrichment analysis of our 13 reproducible proteins and taking the top 50 neighbours into account.

A total of 111 proteins were identified as associated with AF, with 84 positively and 27 negatively correlated with AF incidence (Table S3). Bioinformatics enrichment analyses revealed that these proteins are involved in key biological processes such as cell adhesion, stress response and extracellular matrix (ECM) organisation (Figure 2A,B). ECM formation emerged as a dominant theme, supported by KEGG pathway enrichment in ECM–receptor interaction and focal adhesion, GO–CC terms related to extracellular matrix structural components, Reactome pathways on ECM organisation (Figure S1), and WikiPathways in focal adhesion (Figure S2).

PPI network analysis identified interleukin‐6 (IL‐6), matrix metalloproteinase‐2 (MMP2), C‐reactive protein (CRP), insulin‐like growth factor 1 (IGF1) and osteopontin (SPP1) as the top five most interactive proteins. These findings suggest that these proteins may play central regulatory roles in the pathogenesis of AF, highlighting their potential as key biomarkers or therapeutic targets (Figure 2C).

### 3.4 Reproducible Proteins Across Studies and Bioinformatics Analysis

**FIGURE 3:**
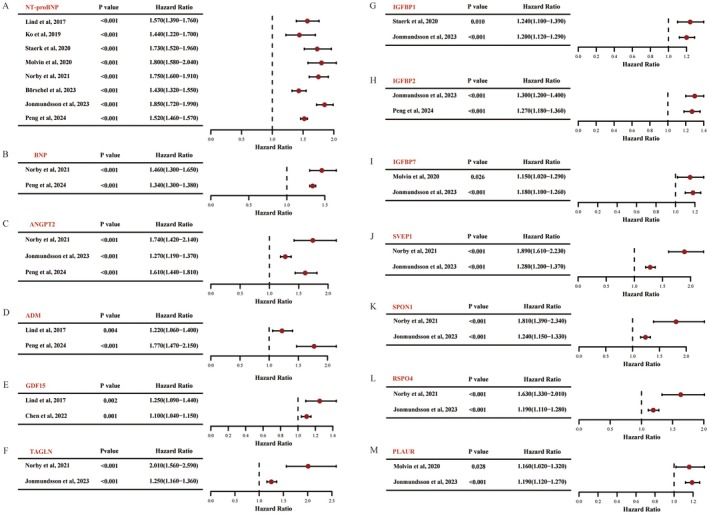
Associations of reproducible proteins with incidence of AF across cohorts. Forest plots showing hazard ratios (HRs) and 95% confidence intervals (CIs) for 13 proteins that were reproducibly associated with AF incidence in at least two cohort studies. Each panel (A–M) represents one protein, with HRs derived from fully adjusted Cox regression models reported in the original studies.

A total of 13 proteins were identified as reproducible across at least two cohorts: N‐terminal pro‐B‐type natriuretic peptide (NT‐proBNP), angiopoietin‐2 (ANGPT‐2), B‐type natriuretic peptide (BNP), adrenomedullin (ADM), growth differentiation factor 15 (GDF15), insulin‐like growth factor‐binding protein 1 (IGFBP‐1), IGFBP‐2, IGFBP‐7, plasminogen activator urokinase receptor (PLAUR, or uPAR), R‐spondin‐4 (RSPO4), Sushi, von Willebrand factor type A, EGF and pentraxin domain‐containing protein 1 (SVEP1), transgelin (TAGLN) and spondin‐1 (SPON1). The strength of association between these reproducible proteins and incidence of AF across individual studies is detailed in Figure 3. NT‐proBNP was reported in eight studies, emerging as the most robust biomarker (Figure 2D). Importantly, the direction of change for these proteins was consistent across all studies, with no conflicting patterns or contradictory findings.

Given the small number of reproducible proteins, network topology‐based enrichment analysis was conducted using the BioGRID PPI network, which incorporated the top 50 neighbouring nodes for GO analysis (12 genes were included as BNP and NT‐proBNP share the same gene, NPPB). The top 10 enriched GO terms (Figure 2E) were related to cell proliferation, signal transduction and growth regulation, suggesting that ECM components may act as scaffolds or signalling mediators in these processes.

### 3.5 Drug Target Analysis of Reproducible Proteins

Drug target analysis revealed that four plasma proteins (ANGPT‐2, ADM, GDF15 and PLAUR) are classified as successful targets or are under investigation in clinical trials, according to the Therapeutic Target Database (Table S4). Notably, drugs targeting GDF15 are currently being evaluated for the treatment of heart failure (NCT05492500). Additionally, the Drug–Gene Interaction Database and DrugBank identified 69 and 13 drug‐target pairs, respectively, involving 10 of the reproducible proteins (Tables S5 and S6). The Open Targets Platform reported 59 clinical trial entries related to ADM and ANGPT‐2 (Table S7).

### 3.6 MR Analysis of Reproducible Proteins

Using cis‐pQTLs as instrumental variables, PWAS analysis did not support a causal relationship between the 13 proteins and AF, atrial structure, or atrial function, as none of the proteins reached the Bonferroni‐corrected significance threshold (Table S8). TWAS analysis, mapping the 13 proteins to 12 encoding genes, revealed a significant negative association between IGFBP‐7 gene expression and AF risk in the eQTLGen dataset (OR = 0.87, 95% CI: 0.81–0.93, *p* = 3.14 × 10^−5^), which contrasts with the plasma IGFBP‐7 levels reported in the included studies. This discrepancy may reflect a potential off‐target effect. Additionally, IGFBP‐7 showed strong colocalisation with AF (PPH4 > 0.8, Table S10), further suggesting a genetic link.

## 4 Discussion

To our knowledge, this is the first systematic review to comprehensively evaluate circulating proteomics studies related to AF incidence. We systematically analysed cohort studies and identified 111 AF‐associated proteins, including 13 reproducible across multiple cohorts, highlighting the potential of proteomic biomarkers for risk assessment and therapeutic exploration. Consistent findings among these biomarkers across studies suggest their promise in improving AF prediction and understanding its pathogenesis.

### 4.1 High‐Throughput Proteomics for Biomarker and Target Discovery

High‐throughput proteomics has revolutionised biomarker discovery and mechanism research in complex diseases like AF by enabling the simultaneous quantification of thousands of circulating proteins. Advanced platforms such as Olink and SomaScan overcome historical technological limitations, offering high sensitivity and specificity for analysing stored biosamples from large‐scale cohorts [43, 44]. These tools bridge clinical and molecular research by identifying disease‐associated proteins and uncovering their roles in key biological pathways, such as extracellular matrix remodelling and signal transduction, supporting precision medicine in AF.

In our study, seven of nine included investigations utilised these advanced platforms, underscoring their widespread adoption. For instance, Jonmundsson et al. [16] employed the SomaScan 7k platform to quantify over 7000 proteins, highlighting the depth of insights achievable through modern proteomics. By facilitating the exploration of biological pathways and leveraging long‐term cohort data, these advancements enhance our understanding of AF pathogenesis and pave the way for improved risk stratification and therapeutic innovation.

### 4.2 Quality of Included Studies and Cross‐Study Comparisons

The included studies were generally of good methodological quality, with most using prospective cohort designs, appropriate confounder adjustments and validated proteomic platforms. Several studies were based on well‐established population cohorts, enhancing the credibility of their findings.

Some heterogeneity in reporting and methodology was noted. For example, not all studies provided standardised protein identifiers (e.g., UniProt IDs), and differences existed in proteomic platforms and quantification approaches. While such variability reflects the evolving nature of proteomic research, it underscores the need for more consistent reporting and methodological alignment. To improve comparability, future studies should adopt more harmonised objectives and standardised reporting frameworks. These efforts will enhance reproducibility and support the integration of proteomic data into AF risk assessment and mechanistic understanding [45].

### 4.3 Key Proteins and Mechanisms Related to AF Incidence

Our analysis identified several key proteins associated with AF incidence, offering insights into the underlying biological mechanisms. These proteins are involved in pathways such as ECM remodelling, inflammation and vascular regulation, which are central to AF pathogenesis.

NT‐proBNP and BNP are released in response to cardiac wall stress and increased pressure load, and they are well‐established biomarkers for assessing HF risk and prognosis [46]. NT‐proBNP, due to its greater stability, is particularly reliable as an indicator of BNP activity. In our analysis, NT‐proBNP emerged as the most robust biomarker associated with AF, consistently identified across nearly all cohorts, thus validating previous findings that NT‐proBNP can predict AF in both the general population and clinical settings [47]. This underscores the adverse impact of increased cardiac load on AF development and highlights it as a key underlying trigger. However, MR analysis did not provide strong evidence of a causal relationship between NT‐proBNP levels and AF, aligning with findings from the FINRISK cohort [48] and AGES‐Reykjavik study [16], which similarly ruled out a causal link between natriuretic peptides and AF incidence at the community level. These results suggest that elevated NT‐proBNP is more likely a marker of underlying structural cardiac changes rather than a direct causal factor in AF pathogenesis. Nevertheless, given its consistent association with AF, NT‐proBNP remains a valuable predictive biomarker for assessing AF risk.

ANGPT‐2 plays a critical role in vascular remodelling by increasing endothelial permeability and promoting inflammatory cell infiltration. It is thought to mediate inflammatory communication between endothelial cells and cardiomyocytes in AF [49]. In the recently published EAST‐AFNET 4 biomolecular study, ANGPT‐2 was shown to predict sinus rhythm maintenance in AF patients, regardless of whether they received rhythm control therapy [50]. These findings underscore the significance of ANGPT‐2 in both the initiation and progression of AF, highlighting its potential as a marker for atrial health. These observations suggest that further research into ANGPT‐2‐regulated atrial disease processes could reveal therapeutic opportunities.

GDF15 is involved in regulating inflammatory responses and cellular stress, both of which are known to be associated with AF [51]. The relationship between GDF15 and AF prognosis has been well confirmed in multiple studies [52, 53, 54], and it has been incorporated into the ABC‐Bleeding risk score to evaluate bleeding risk in AF patients undergoing anticoagulation therapy [55]. However, findings on the association between GDF15 and AF incidence have been inconsistent. For instance, in the Akershus Cardiac Examination 1950 Study, GDF15 was not significantly associated with AF after adjustment for confounders, and it did not show prognostic value for AF incidence [56]. In contrast, a MR analysis indicated that elevated circulating GDF15 levels were significantly associated with increased AF risk (OR = 1.03, *p* = 0.043) [51]. These findings suggest that, while GDF15 may be elevated in AF patients, it is more likely reflective of a broader inflammatory state or cardiac stress, rather than actively contributing to atrial remodelling or the electrophysiological changes that trigger AF. Thus, although GDF15 remains a marker of cardiovascular risk, its utility in AF‐specific risk prediction appears limited, and further research is needed to elucidate its role in atrial pathophysiology.

IGFBPs are involved in regulating cell growth, apoptosis, and extracellular matrix stability, processes relevant to AF pathogenesis [57]. In particular, IGFBP‐2 has shown a significant positive correlation with left atrial volume index, an association that has been validated in two independent cohorts [58]. This suggests that IGFBP‐2 may be involved in atrial structural changes, particularly through pathways related to fibrosis. Elevated levels of IGFBP‐7 have also been linked to AF risk, although MR analysis indicated a potential protective role, which contrasts with the observed plasma associations. Such discrepancies highlight the complex interactions within the IGF pathway, where context‐dependent effects may influence the overall risk for atrial remodelling and AF. Thus, IGFBPs, particularly IGFBP‐2, appear to play a role in atrial structural alterations, potentially contributing to the pro‐fibrotic substrate that predisposes to AF.

In addition to the reproducible proteins identified across cohorts, several other proteins with plausible mechanistic relevance to AF have been reported in the literature, although they were not consistently detected in multiple studies included in our review. One such protein is von Willebrand factor (vWF), a well‐known endothelial glycoprotein involved in haemostasis and vascular integrity [59]. Elevated vWF levels have been reported in patients with nonvalvular AF and linked to progression to permanent AF despite anticoagulation [60, 61]. Mechanistically, vWF may promote atrial remodelling through oxidative stress and endothelin‐1 signalling [62]. In experimental models, endothelial knockdown of vWF suppressed Ang II‐induced endothelin‐1 expression and reduced NOX‐mediated oxidative stress, implicating vWF in endothelial dysfunction during AF [63]. These findings suggest that vWF may not only reflect endothelial injury but also contribute to AF pathophysiology and adverse outcomes.

The MR analysis of these key proteins generally suggests that while many of these proteins are strongly associated with AF risk in observational studies, their causal role in AF pathogenesis remains unclear. This may indicate that these proteins serve more as markers of underlying pathophysiological processes—such as atrial remodelling, inflammation, or vascular dysfunction—rather than as direct contributors to AF onset. The discrepancies between MR and observational findings also underscore the complexity of AF as a multifactorial disease, involving diverse pathways and interlinked biological processes. Further research into these proteins may not only provide deeper insights into the biological pathways that drive AF but also identify potential therapeutic targets for preventing or mitigating atrial remodelling. A better understanding of these mechanisms could facilitate the development of new therapies aimed at targeting the molecular underpinnings of AF, ultimately improving patient outcomes.

### 4.4 Challenges and Future Directions for Integrating Proteomics Into AF Risk Stratification

Our systematic review identified several proteins reproducibly associated with AF risk, such as NT‐proBNP, ANGPT2 and GDF15, across diverse cohorts and proteomic platforms. These consistent associations underscore the potential of circulating proteomics to complement traditional risk factors and enhance our understanding of AF pathophysiology.

Current guidelines, including the 2023 ACC/AHA/ACCP/HRS and 2024 ESC AF guidelines, acknowledge the prognostic utility of natriuretic peptides and inflammatory biomarkers, but integration into routine AF screening remains limited [5, 64]. This is partly due to the multifactorial nature of AF, involving fibrosis, inflammation and metabolic dysregulation. Single biomarkers may not fully capture this complexity, which reinforces the need for more comprehensive approaches.

Emerging strategies such as composite biomarker panels and hub‐protein‐based scoring systems hold great promise. Insights from other fields, like Alzheimer's disease research [65], suggest that high‐throughput proteomics combined with machine learning and network analysis can uncover pathophysiologically relevant clusters of proteins. Applying similar approaches in AF could improve both prediction and mechanistic insight, enabling more personalised prevention strategies.

To move toward clinical implementation, future studies should focus on validating biomarker panels in large, diverse cohorts, establishing standardised proteomic protocols, and exploring optimal measurement frequencies. While challenges related to logistics and cost remain, ongoing advances in proteomics and data science offer solutions. With coordinated efforts, proteomics‐based risk models could become valuable tools in the personalised management of AF.

### 4.5 Origins and Modifiable Influences of AF‐Related Proteins

As discussed above, proteomics has enabled the identification of circulating proteins associated with AF, providing valuable insights for risk prediction and mechanistic exploration. However, increasing attention is now being paid to the biological origins, distribution, transport and regulation of these proteins within the body. Traditionally, circulating proteins were thought to be primarily secreted by organs and tissues such as the heart, liver, blood vessels and immune cells [66]. Recent studies, however, have highlighted the pivotal role of exosomes and other extracellular vesicles in mediating intercellular protein transport and signalling [67, 68]. For example, AF‐related proteins like GDF15 can be actively packaged into exosomes, which then deliver these molecules via the bloodstream to distant target tissues, facilitating complex cell‐to‐cell communication [69]. This not only adds a new layer of diversity to protein signalling regulation but also provides novel biological explanations for the onset and progression of AF. Moreover, exosome‐mediated delivery can enhance protein stability, alter biological activity, and influence target specificity, potentially leading to differences in protein expression profiles between biofluids and tissues [70]. Therefore, when interpreting plasma proteomics data, it is essential to consider both the cellular origin of proteins and their transport mechanisms.

In addition to endogenous factors, external exposures and lifestyle choices also significantly influence the circulating proteome [71]. Diet, medications, physical activity, and metabolic status can all modulate the synthesis, secretion and degradation of proteins [72]. Recent clinical evidence indicates that regular omega‐3 fatty acid supplementation may increase AF risk through the upregulation of Piezo1 protein activity, highlighting the complex interplay between nutritional factors and circulating proteomics [73]. Such findings emphasise the importance of considering dietary patterns, medication exposure, and other modifiable factors when interpreting the relationship between protein biomarkers and AF risk.

More broadly, the expression and transport of circulating proteins are shaped not only by genetic and disease factors but also by the environment and lifestyle. Future research should integrate the study of protein origin, extracellular vesicle‐mediated transport, and exogenous modulators such as diet and medication into comprehensive proteomic frameworks. Only by considering these multi‐level influences can we more accurately evaluate the clinical significance of protein biomarkers and advance the translation of proteomic findings into precise AF risk stratification and individualised prevention strategies.

### 4.6 Strengths and Limitations

Our study has several strengths, including the inclusion of globally recognised cohort studies, many of which have generated high‐quality results, enhancing the credibility and representativeness of our findings. However, some limitations should be acknowledged. First, the study population was predominantly of European ancestry, limiting the generalisability of our results to other ethnic groups. Second, differences in sequencing methods and protein spectra across the included studies may have introduced biases, potentially overemphasised well‐known proteins while overlooking others critical to AF pathogenesis. Third, inconsistencies in sample sizes, follow‐up durations, and covariate adjustments, as well as reliance on ECG for AF diagnosis, may have affected the accuracy of regression analyses, particularly given the latent nature of AF and the potential for underdiagnosis. Fourth, this study relied on aggregated data, restricting our ability to analyse individual‐level associations. Individual‐level analyses would offer more granular insights into protein‐AF relationships. Finally, the Mendelian randomisation analysis was limited by the availability of pQTLs and eQTLs and was potentially affected by horizontal pleiotropy, necessitating further validation of the identified causal relationships. Despite these limitations, our study highlights the utility of proteomics in AF research and underscores the need for future investigations to address these challenges and expand the generalisability of findings.

## 5 Conclusion

This study underscores the potential of high‐throughput proteomics in identifying biomarkers and unravelling mechanisms underlying AF. While reproducible proteins such as NT‐proBNP and ANGPT‐2 highlight important pathways like ECM remodelling and inflammation, their causal roles remain uncertain. Future efforts should focus on integrating composite biomarker panels and standardising methodologies to enhance clinical applicability and improve AF risk prediction.

## Author Contributions

**Luxiang Shang:** data curation (equal), funding acquisition (equal), investigation (equal), resources (equal), software (equal), writing – original draft (equal). **Weilin Wang:** data curation (equal), methodology (equal), software (equal). **Yiying Liu:** conceptualization (equal), resources (equal), software (equal). **Baopeng Tang:** data curation (equal), validation (equal), writing – review and editing (equal). **Yinglong Hou:** conceptualization (equal), funding acquisition (equal), writing – review and editing (equal).

## Conflicts of Interest

The authors declare no conflicts of interest.

## Supporting information


**Appendix S1:** jcmm70760‐sup‐0001‐AppendixS1.docx.

## Data Availability

All data used in this study were included in the manuscript and Supporting Information.
