# Supplementary material for: Circulating Proteomics and Risk of Atrial Fibrillation: A Systematic Review of Cohort Studies
Source: J Cell Mol Med. 2025 Aug 5;29(15):e70760. doi: 10.1111/jcmm.70760 (PMC12324954; doi:10.1111/jcmm.70760)
Supplement: Supplementary file 1 — Appendix S1: jcmm70760‐sup‐0001‐AppendixS1.docx. [file JCMM-29-e70760-s001.docx]

**Circulating proteomics and risk of atrial fibrillation: A systematic review of cohort studies**

**Supplementary files:**

Supplementary Table S1. Complete search strategy and results.

Supplementary Table S2. Data sources in the MR analysis.

Supplementary Table S3. UniProt accession number, protein name, gene name and class of proteins associated with incident AF.

Supplementary Table S4. Therapeutic Target Database for the reproducible proteins.

Supplementary Table S5. Drug Gene interaction Database for the reproducible proteins.

Supplementary Table S6. DrugBank database for the reproducible proteins.

Supplementary Table S7. Open Targets for the reproducible proteins.

Supplementary Table S8. Genetic association of 13 reproducible proteins on AF and atrial traits.

Supplementary Table S9. Effect of plasma and tissue-specific protein-coding gene expression on AF risk for the 13 reproducible proteins.

Supplementary Table S10. Results of colocalization of the 13 reproducible proteins with AF.

Supplementary Figure S1. Enrichment analysis of all AF-related proteins based on Reactome.

Supplementary Figure S2. Enrichment analysis of all AF-related proteins based on WikiPathways.

**Supplementary Table S1. Complete search strategy and results**

| **Database** | **Search queries** | **Terms** | **Results (Nov 4, 2024)** |
| --- | --- | --- | --- |
| **PubMed** | #1 | (atrial fibrillation[MeSH Terms] OR atrial fibrillation[Title/abstract]) | 115,555 |
|  | #2 | (“Proteomics”[Mesh] OR “Proteome”[Mesh] OR proteom*[tiab] OR “aptamer based”[tiab] OR “antibody based”[tiab] OR “aptamer-based”[tiab] OR “antibody-based”[tiab] OR somalogic[tiab] OR Luminex[tiab] OR Olink[tiab] OR “o link”[tiab] OR “serum marker”[tiab] OR “serum markers”[tiab] OR “serum proteome*”[tiab] OR “serum proteomics”[tiab] OR “serum biomarkers”[tiab] OR “serum biomarker”[tiab] OR “plasma proteome*”[tiab] OR “plasma proteomics”[tiab] OR “plasma marker”[tiab] OR “plasma markers”[tiab] OR “plasma biomarker”[tiab] OR “plasma biomarkers”[tiab] OR “prote-omic*”[tiab] OR “protein omic*”[tiab] OR proteinomic*[tiab]) | 204,200 |
|  | #3 | (prospective[Title/abstract] OR follow-up[Title/abstract] OR cohort[Title/abstract] OR longitudinal[Title/abstract] OR nested case-control[Title/abstract]) | 2,748,339 |
|  | #4 | #1 and #2 and #3 not (animals[MeSH Terms] NOT humans[MeSH Terms]) and (“2010”[Date – Publication] : “2024”[Date – Publication]) | 88 |
|  | #5 | #4 not (editorial[Publication Type] OR comment[Publication Type] OR news[Publication Type] OR “Congress”[Publication Type] OR “Consensus Development Conference”[Publication Type] OR editorial[tiab] OR commentary[tiab] OR “conference abstract*”[tiab] OR “conference proceeding*”[tiab] OR symposium*[tiab] OR “Published Erratum”[Publication Type] OR errata[tiab] OR erratum[tiab] OR corrigenda[tiab] OR corrigendum[tiab] OR protocol[ti] OR protocols[ti] OR “Review”[Publication Type] OR “systematic review*”[tiab] OR “Systematic Review”[Publication Type] OR “integrative review*”[tiab]) | 78 |
| **Embase** | #1 | (‘atrial fibrillation’/exp OR ‘atrial fibrillation’:ti,ab) | 250,329 |
|  | #2 | (‘proteomics’/exp OR ‘proteome’/de OR proteom*:ti,ab OR ‘aptamer based’:ti,ab OR ‘antibody based’:ti,ab OR ‘aptamer-based’:ti,ab OR ‘antibody-based’:ti,ab OR somalogic:ti,ab OR Luminex:ti,ab OR Olink:ti,ab OR ‘o link’:ti,ab OR ‘serum marker’:ti,ab OR ‘serum markers’:ti,ab OR ‘serum proteome*’:ti,ab OR ‘serum proteomics’:ti,ab OR ‘serum biomarkers’:ti,ab OR ‘serum biomarker’:ti,ab OR ‘plasma proteome*’:ti,ab OR ‘plasma proteomics’:ti,ab OR ‘plasma marker’:ti,ab OR ‘plasma markers’:ti,ab OR ‘plasma biomarker’:ti,ab OR ‘plasma biomarker*’:ti,ab OR ‘prote-omic*’:ti,ab OR ‘protein omic*’:ti,ab OR proteinomic*:ti,ab) | 286,802 |
|  | #3 | (‘prospective’:ti,ab OR ‘follow-up’:ti,ab OR ‘cohort’:ti,ab OR ‘longitudinal’:ti,ab OR ‘nested case-control’:ti,ab) | 4,261,617 |
|  | #4 | #1 AND #2 AND #3 AND [humans]/lim AND [2010-2024]/py | 222 |
|  | #5 | #4 NOT ([conference abstract]/lim OR [conference paper]/lim OR [conference review]/lim OR [data papers]/lim OR [editorial]/lim OR [erratum]/lim OR [note]/lim OR [review]/lim OR [short survey]/lim OR [systematic review]/lim OR ‘data paper’/exp OR ‘editorial’/exp OR ‘erratum’/exp OR ‘note’/exp OR ‘short survey’/exp OR ‘review’/exp OR ‘systematic review’/exp OR corrigenda:ti,ab OR corrigendum:ti,ab OR protocol:ti OR protocols:ti OR erratum:ti,ab OR errata:ti,ab OR ‘conference abstract*’:ti,ab OR ‘conference proceeding*’:ti,ab OR symposium*:ti,ab OR editorial:ti,ab OR commentary:ti,ab OR ‘systematic review*’:ti,ab OR ‘integrative review*’:ti,ab) | 130 |
| **Web of Science: Core Collection** | #1 | TS=“atrial fibrillation” | 118,232 |
|  | #2 | TS=((proteom* OR "aptamer based" OR "antibody based" OR "aptamer-based" OR "antibody-based" OR somalogic OR Luminex OR Olink OR "o link" OR "serum marker" OR "serum markers" OR "serum proteome*" OR "serum proteomics" OR "serum biomarkers" OR "serum biomarker" OR "plasma proteome*" OR "plasma proteomics" OR "plasma marker" OR "plasma markers" OR "plasma biomarker" OR "plasma biomarkers" OR "prote-omic*" OR "protein omic*" OR proteinomic*)) | 220,500 |
|  | #3 | TS=(prospective OR follow-up OR cohort OR longitudinal OR nested case-control) | 2,841,618 |
|  | #4 | #1 and #2 and #3 and PY=(2010-2024) | 97 |
|  | #5 | #4 not TI=(editorial OR commentary OR "conference abstract*" OR "conference proceeding*" OR symposium* OR errata OR erratum OR corrigenda OR corrigendum OR protocol OR protocols OR "systematic review*" OR "integrative review*") NOT DT=(Editorial Material OR News Item OR Note OR Book OR Book Chapter OR Excerpt OR Item About an Individual OR Meeting Abstract OR Meeting Summary OR Reprint) | 93 |

**Supplementary Table S2. Data sources in the MR analysis**

| Characteristic | Data source | Sample size (cases/controls) | Population ancestry | PMID/website |
| --- | --- | --- | --- | --- |
| **pQTL data** |  |  |  |  |
| proteins | deCODE | 35,559 | European | 34857953 |
| **eQTL data** |  |  |  |  |
| eQTL in eQTLGen | eQTLGen Consortium | Whole blood: 31684 | Predominantly European | https://www.eqtlgen.org/cis-eqtls.html |
| eQTL in GTEx | GTEx V8 | Whole blood: 670 Left ventricle: 386 Atrial appendage: 372 | Predominantly European | https://www.gtexportal.org/home/datasets |
| **GWAS summary data** |  |  |  |  |
| Atrial fibrillation | The Nord-Trøndelag Health Study (HUNT), deCODE, the Michigan Genomics Initiative (MGI), DiscovEHR, UK Biobank, and the AFGen Consortium | 60,620/970,216 | European | 30061737 |
| Atrial fibrillation | FinnGen r11 | 55,853/231,952 | European | https://www.finngen.fi/en |
| Left atrial maximum volume (BSA indexed) | UK Biobank | 35,049 | European | 38773065 |
| Left atrial minimum volume (BSA indexed) | UK Biobank | 35,049 | European | 38773065 |
| Left atrial stroke volume (BSA indexed) | UK Biobank | 35,049 | European | 38773065 |
| Left atrial emptying fraction | UK Biobank | 35,049 | European | 38773065 |

**Supplementary Table S3. UniProt accession number, protein name, gene name and class of proteins associated with incident AF**

| AF-positive associated protein | | | | |
| --- | --- | --- | --- | --- |
| Source | UniProt ID | Protein name | Gene name | PANTHER protein class |
| Lind et al, 2017 | P16860 | N-terminal pro-B-type natriuretic peptide (NT-proBNP) | NPPB | No class assigned |
|  | Q9GZV9 | Fibroblast growth factor 23 | FGF23 | Growth factor |
|  | P05231 | Interleukin-6 | IL6 | No class assigned |
|  | P15090 | Fatty acid-binding protein 4 | FABP4 | Transfer/carrier protein |
|  | Q99988 | Growth differentiation factor 15 (GDF15) | GDF15 | Growth factor |
|  | Q96D42 | T-cell immunoglobulin and mucin domain 1 | HAVCR1 | No class assigned |
|  | P35318 | Adrenomedullin | ADM | Peptide hormone |
| Ko et al, 2019 | P16860 | NT-proBNP | NPPB | No class assigned |
| Staerk et al, 2020 | P16860 | NT-proBNP | NPPB | No class assigned |
|  | P08833 | Insulin-like growth factor-binding protein 1 | IGFBP1 | Protease inhibitor |
| Molvin et al, 2020 | P16860 | NT-proBNP | NPPB | No class assigned |
|  | P08253 | Matrix metalloproteinase-2 | MMP2 | Metalloprotease |
|  | Q03405 | Urokinase plasminogen activator surface receptor | PLAUR | Transmembrane signal receptor |
|  | P10451 | Osteopontin | SPP1 | Cytokine |
|  | Q16270 | Insulin-like growth factor-binding protein 7 | IGFBP7 | No class assigned |
| Norby et al, 2021 | P16860 | NT-proBNP | NPPB | No class assigned |
|  | Q4LDE5 | Sushi, von Willebrand factor type A, EGF and pentraxin domain-containing protein 1 | SVEP1 | Complement component |
|  | P16860 | Natriuretic peptides B | NPPB | No class assigned |
|  | Q01995 | Transgelin | TAGLN | No class assigned |
|  | O15123 | Angiopoietin-2 | ANGPT2 | Intercellular signal molecule |
|  | O94813 | Slit homolog 2 protein | SLIT2 | No class assigned |
|  | Q496F6 | CMRF35-like molecule 2 | CD300E | Immunoglobulin receptor superfamily |
|  | P03973 | Antileukoproteinase | SLPI | Protease inhibitor |
|  | P21815 | Bone sialoprotein 2 | IBSP | Extracellular matrix structural protein |
|  | P55083 | Microfibril-associated glycoprotein 4 | MFAP4 | Intercellular signal molecule |
|  | Q5BIV9 | Shadow of prion protein | SPRN | No class assigned |
|  | Q2I0M5 | R-spondin-4 | RSPO4 | No class assigned |
|  | Q9BU40 | Chordin-like protein 1 | CHRDL1 | No class assigned |
|  | Q9HCB6 | Spondin-1 | SPON1 | Cell adhesion molecule |
| Chen et al, 2022 | Q99988 | GDF15 | GDF15 | Growth factor |
| Börschel et al, 2023 | P16860 | NT-proBNP | NPPB | No class assigned |
| Jonmundsson et al, 2023 | P16860 | NT-proBNP | NPPB | No class assigned |
|  | Q4LDE5 | Sushi, von Willebrand factor type A, EGF and pentraxin domain-containing protein 1 | SVEP1 | Complement component |
|  | O15123 | Angiopoietin‐2 | ANGPT2 | Intercellular signal molecule |
|  | P18065 | Insulin-like growth factor-binding protein 2 | IGFBP2 | Protease inhibitor |
|  | Q13790 | Apolipoprotein F | APOF | Apolipoprotein |
|  | Q9H0R8 | Gamma-aminobutyric acid receptor-associated protein-like 1 | GABARAPL1 | Non-motor microtubule binding protein |
|  | Q7LFX5 | Carbohydrate sulfotransferase 15 | CHST15 | Transferase |
|  | Q9HCB6 | Spondin-1 | SPON1 | Cell adhesion molecule |
|  | O43915 | Vascular endothelial growth factor D | VEGFD | Growth factor |
|  | Q03405 | Urokinase plasminogen activator surface receptor | PLAUR | Transmembrane signal receptor |
|  | Q99645 | Epiphycan | EPYC | No class assigned |
|  | Q76M96 | Coiled-coil domain-containing protein 80 | CCDC80 | No class assigned |
|  | P42167 | amina-associated polypeptide 2, isoforms beta/gamma | TMPO | Peptide hormone |
|  | Q15848 | Adiponectin | ADIPOQ | Scaffold/adaptor protein |
|  | Q01995 | Transgelin | TAGLN | No class assigned |
|  | P61769 | Beta-2-microglobulin | B2M | Major histocompatibility complex protein |
|  | Q13219 | Pappalysin-1 | PAPPA | No class assigned |
|  | P01034 | Cystatin-C | CST3 | Protease inhibitor |
|  | P23381 | Tryptophan--tRNA ligase, cytoplasmic | WARS | Aminoacyl-trna synthetase |
|  | Q9NQ30 | Endothelial cell-specific molecule 1 | ESM1 | No class assigned |
|  | P21246 | Pleiotrophin | PTN | Growth factor |
|  | Q14508 | WAP four-disulfide core domain protein 2 | WFDC2 | Protease inhibitor |
|  | O95715 | C-X-C motif chemokine 14 | CXCL14 | Cytokine |
|  | P19429 | Troponin I, cardiac muscle | TNNI3 | Non-motor actin binding protein |
|  | O95633 | Follistatin-related protein 3 | FSTL3 | Protease inhibitor |
|  | Q5JZY3 | Ephrin type-A receptor 10 | EPHA10 | Transmembrane signal receptor |
|  | P68402 | Platelet-activating factor acetylhydrolase IB subunit alpha2 | PAFAH1B2 | Protein modifying enzyme |
|  | P08833 | Insulin-like growth factor-binding protein 1 | IGFBP1 | Protease inhibitor |
|  | O75339 | Cartilage intermediate layer protein 1 | CILP | No class assigned |
|  | Q2I0M5 | R-spondin-4 | RSPO4 | No class assigned |
|  | Q16270 | Insulin-like growth factor-binding protein 7 | IGFBP7 | No class assigned |
|  | P35442 | Thrombospondin-2 | THBS2 | Cell adhesion molecule |
|  | Q9UMF0 | Intercellular adhesion molecule 5 | ICAM5 | Cell adhesion molecule |
|  | P27348, P31947, P31946, P62258, P61981, Q04917, P63104 | 14-3-3 protein theta, 14-3-3 protein sigma, 14-3-3 protein beta/alpha, 14-3-3 protein epsilon, 14-3-3 protein gamma, 14-3-3 protein eta, 14-3-3 protein zeta/delta | YWHAQ;SFN;YWHAB;YWHAE;YWHAG;YWHAH;YWHAZ | Scaffold/adaptor protein |
|  | P48061 | Stromal cell-derived factor 1 | CXCL12 | Cytokine |
|  | P54826 | Growth arrest-specific protein 1 | GAS1 | No class assigned |
|  | Q9BXY4 | R-spondin-3 | RSPO3 | No class assigned |
|  | P04632, P07384 | Calpain I | CAPN1;CAPNS1 | Cysteine protease |
|  | Q08708 | CMRF35-like molecule 6 | CD300C | Immunoglobulin receptor superfamily |
|  | Q15063 | Periostin | POSTN | Cell adhesion molecule |
|  | Q8N474 | Secreted frizzled-related protein 1 | SFRP1 | Transmembrane signal receptor |
|  | Q9H4F8 | SPARC-related modular calcium-binding protein 1 | SMOC1 | Calmodulin-related |
|  | O14896 | Interferon regulatory factor 6 | IRF6 | Winged helix/forkhead transcription factor |
|  | Q9NPY3 | Complement component C1q receptor | CD93 | Extracellular matrix structural protein |
|  | P14555 | Phospholipase A2, membrane associated | PLA2G2A | Phospholipase |
|  | Q9H773 | dCTP pyrophosphatase 1 | DCTPP1 | Phosphatase |
|  | Q96F46 | Interleukin-17 receptor A | IL17RA | Transmembrane signal receptor |
|  | P39060 | Collagen alpha-1(XVIII) chain | COL18A1 | Extracellular matrix structural protein |
|  | Q9BQT9 | Calsyntenin-3 | CLSTN3 | Membrane traffic protein |
|  | P02748 | Complement component C9 | C9 | Complement component |
|  | Q8N2S1 | Latent-transforming growth factor beta-binding protein 4 | LTBP4 | Extracellular matrix structural protein |
|  | P00746 | Complement factor D | CFD | Serine protease |
|  | P13987 | CD59 glycoprotein | CD59 | No class assigned |
|  | O60330 | Protocadherin gamma-A12 | PCDHGA12 | Cadherin |
|  | P42224 | Signal transducer and activator of transcription 1-alpha/beta | STAT1 | Rel homology transcription factor |
|  | Q07325 | C-X-C motif chemokine 9 | CXCL9 | Cytokine |
|  | P02741 | C-reactive protein | CRP | Actin or actin-binding cytoskeletal protein |
| Peng et al, 2024 | P16860 | NT-proBNP | NPPB | No class assigned |
|  | P16860 | Natriuretic peptides B | NPPB | No class assigned |
|  | P02462 | Collagen alpha-1(IV) chain | COL4A1 | extracellular matrix structural protein |
|  | P18065 | Insulin-like growth factor-binding protein 2 | IGFBP2 | protease inhibitor |
|  | P39060 | Collagen alpha-1(XVIII) chain | COL18A1 | extracellular matrix structural protein |
|  | P24821 | Tenascin | TNC | extracellular matrix protein |
|  | P98160 | Basement membrane-specific heparan sulfate proteoglycan core protein | HSPG2 | immunoglobulin superfamily cell adhesion molecule |
|  | P62736 | Actin, aortic smooth muscle | ACTA2 | actin and actin related protein |
|  | P23327 | Sarcoplasmic reticulum histidine-rich calcium-binding protein | HRC | No class assigned |
|  | P05305 | Endothelin-1 | EDN1 | peptide hormone |
|  | Q9H4D0 | Calsyntenin-2 | CLSTN2 | membrane traffic protein |
|  | P19883 | Follistatin | FST | protease inhibitor |
|  | P13747 | HLA class I histocompatibility antigen, alpha chain E | HLA-E | major histocompatibility complex protein |
|  | O15123 | Angiopoietin-2 | ANGPT2 | intercellular signal molecule |
|  | P35318 | Pro-adrenomedullin | ADM | peptide hormone |
|  | Q9NP84 | Tumor necrosis factor receptor superfamily member 12A | TNFRSF12A | transmembrane signal receptor |

| AF-negative associated protein | | | | |
| --- | --- | --- | --- | --- |
| Source | UniProt accession | Protein name | Gene name | PANTHER protein class |
| Ko et al, 2019 | Q76LX8 | A disintegrin and metalloproteinase with thrombospondin motifs 13 | ADAMTS13 | Metalloprotease |
| Staerk et al, 2020 | P05019 | Insulin-like growth factor I | IGF1 | Growth factor |
| Norby et al, 2021 | P80370 | Protein delta homolog 1 | DLK1 | Membrane-bound signaling molecule |
| Jonmundsson et al, 2023 | P04070 | Vitamin K-dependent protein C | PROC | Serine protease |
|  | P08185 | Corticosteroid-binding globulin | SERPINA6 | Protease inhibitor |
|  | P61024 | Cyclin-dependent kinases regulatory subunit 1 | CKS1B | Scaffold/adaptor protein |
|  | Q14289 | Protein-tyrosine kinase 2-beta | PTK2B | Non-receptor tyrosine protein kinase |
|  | Q15750, O43318 | Mitogen-activated protein kinase kinase kinase 7, TGF-beta-activated kinase 1 and MAP3K7-binding protein 1 fusion | TAB1;MAP3K7 | Protein phosphatase |
|  | O75636 | Ficolin-3 | FCN3 | Intercellular signal molecule |
|  | P05026 | Sodium/potassium-transporting ATPase subunit beta-1 | ATP1B1 | Primary active transporter |
|  | Q9UBP0 | Spastin | SPAST | Non-motor microtubule binding protein |
|  | Q8TEU8 | WAP, Kazal, immunoglobulin, Kunitz and NTR domain-containing protein 2 | WFIKKN2 | No class assigned |
|  | P15121 | Aldo-keto reductase family 1 member B1 | AKR1B1 | Reductase |
|  | Q9BXN1 | Asporin | ASPN | No class assigned |
|  | Q15722 | Leukotriene B4 receptor 1 | LTB4R | G-protein coupled receptor |
|  | Q50LG9 | Leucine-rich repeat-containing protein 24 | LRRC24 | Transmembrane signal receptor |
|  | Q8TDY8 | Immunoglobulin superfamily DCC subclass member 4 | IGDCC4 | Cell adhesion molecule |
|  | P20936 | Ras GTPase-activating protein 1 | RASA1 | Gtpase-activating protein |
|  | Q13508 | Ecto-ADP-ribosyltransferase 3 | ART3 | Transferase |
|  | Q8IYJ0 | PILR alpha-associated neural protein | PIANP | No class assigned |
|  | P25391, P07942, P11047 | Laminin subunit alpha-1, Laminin subunit beta-1, Laminin subunit gamma-1 | LAMA1;LAMB1;LAMC1 | Cell adhesion molecule |
|  | Q9Y2C2 | Uronyl 2-sulfotransferase | UST | Transferase |
| Peng et al, 2024 | Q99435 | Protein kinase C-binding protein NELL2 | NELL2 | calcium-binding protein |
|  | P0DUB6_P0DTE7_P0DTE8 | Alpha-amylase 1A, Alpha-amylase 1B, Alpha-amylase 1C | AMY1A_AMY1B_AMY1C | amylase |
|  | Q96GW7 | Brevican core protein | BCAN | extracellular matrix glycoprotein |
|  | Q8N967 | Leucine-rich repeat and transmembrane domain-containing protein 2 | LRTM2 | No class assigned |
|  | P07949 | Proto-oncogene tyrosine-protein kinase receptor Ret | RET | transmembrane signal receptor |

online sources: UniProt (https://www.uniprot.org/) and PANTHER (http://www.pantherdb.org/)

**Supplementary Table S4. Therapeutic Target Database for the reproducible proteins.**

| Gene | Target name | Target type | Disease | Drugs | Drug info |
| --- | --- | --- | --- | --- | --- |
| NPPB | B-type natriuretic peptide (BNP) | Literature-reported Target | Hypertension | BD-NP | https://db.idrblab.net/ttd/data/drug/details/d06mpj |
| ANGPT2 | Angiopoietin-2 (ANGPT2) | Successful Target | Neovascular age-related macular degeneration; Breast cancer | Faricimab | https://db.idrblab.net/ttd/data/drug/details/d9mwl4 |
| ADM | Adrenomedullin (ADM) | Clinical trial Target | Respiratory distress syndrome; Acute respiratory distress syndrome | BAY1097761 | https://db.idrblab.net/ttd/data/drug/details/dk8bs7 |
| GDF15 | Growth/differentiation factor 15 (GDF15) | Clinical trial Target | Cancer cachexia; Heart failure | Ponsegromab | https://db.idrblab.net/ttd/data/drug/details/d97bht |
| IGFBP1 | Insulin-like growth factor-binding protein 1 (IGFBP1) | Literature-reported Target | NA | NA | NA |
| IGFBP2 | Insulin-like growth factor-binding protein 2 (IGFBP2) | Literature-reported Target | NA | NA | NA |
| IGFBP7 | Insulin-like growth factor-binding protein 7 (IGFBP7) | Literature-reported Target | NA | NA | NA |
| PLAUR | Urokinase plasminogen activator surface receptor (PLAUR) | Successful Target | High-risk myelofibrosis; Essential thrombocythemia | Ruxolitinib | https://db.idrblab.net/ttd/data/drug/details/d04lks |
| RSPO4 | NA | NA | NA | NA | NA |
| SVEP1 | NA | NA | NA | NA | NA |
| TAGLN | Transgelin (TAGLN) | Literature-reported Target | NA | NA | NA |
| SPON1 | NA | NA | NA | NA | NA |

**Supplementary Table S5. Drug Gene interaction Database for the reproducible proteins.**

| Gene | Drug | Regulatory approval | Indication |
| --- | --- | --- | --- |
| ADM | PAROXETINE HYDROCHLORIDE, HEMIHYDRATE | Approved | antidepressant |
| ADM | CHEMBL:CHEMBL2063510 | Not Approved |  |
| ADM | INDOMETHACIN | Approved | NSAID |
| ADM | ALTEPLASE | Approved | Thrombolytic agent |
| ADM | PD-98059 | Not Approved |  |
| ADM | CHEMBL:CHEMBL472004 | Not Approved |  |
| ADM | INSULIN, REGULAR, HUMAN | Approved | for treatment of diabetic foot ulcers,antidiabetic |
| ADM | ENIBARCIMAB | Not Approved |  |
| PLAUR | LENOGRASTIM | Approved |  |
| PLAUR | PHORBOL 12-MYRISTATE 13-ACETATE | Not Approved |  |
| PLAUR | RUXOLITINIB | Approved | antiinflammatory agent,antineoplastic agent |
| PLAUR | RECOMBINANT HUMAN MONOCYTE CHEMOATTRACTANT PROTEIN-2 | Not Approved |  |
| PLAUR | RECOMBINANT INTERFERON GAMMA | Not Approved |  |
| PLAUR | ADENOVIRUS VECTOR | Not Approved |  |
| PLAUR | DIPHTHERIA TOXIN | Not Approved |  |
| PLAUR | UROKINASE | Approved | Thrombolytic Agents,thrombolytic agent |
| IGFBP1 | STREPTOZOCIN | Approved |  |
| IGFBP1 | THERAPEUTIC HORMONE | Not Approved |  |
| IGFBP1 | TETRADECANOYLPHORBOL ACETATE | Not Approved |  |
| IGFBP1 | SANDOSTATIN | Not Approved |  |
| IGFBP1 | DEHYDRATED ALCOHOL | Approved |  |
| IGFBP1 | LIOTHYRONINE | Approved | Hormone Replacement Agents |
| IGFBP1 | ORAL CONTRACEPTIVE | Not Approved |  |
| IGFBP1 | PROTEIN SYNTHESIS INHIBITOR | Not Approved |  |
| IGFBP1 | SIROLIMUS | Approved | for treatment of wet age-related macular degeneration,immunosuppressant |
| IGFBP1 | DROLOXIFENE | Not Approved |  |
| IGFBP1 | OCTREOTIDE ACETATE | Approved | antihypertensive agent,antineoplastic agent |
| IGFBP1 | BUSERELIN | Approved |  |
| IGFBP1 | DEXAMETHASONE | Approved | for treatment of Meniere's disease,glucocorticoid,antiinflammatory agent |
| TAGLN | RECOMBINANT TRANSFORMING GROWTH FACTOR-BETA 1 | Not Approved |  |
| TAGLN | AZACITIDINE | Approved | antineoplastic agent |
| SVEP1 | TICAGRELOR | Approved | antithrombotic |
| GDF15 | INDOLE-3-CARBINOL | Not Approved |  |
| GDF15 | ETOPOSIDE | Approved | Antineoplastic Agents |
| GDF15 | SULINDAC SULFIDE | Not Approved |  |
| GDF15 | NONSTEROIDAL ANTIINFLAMMATORY DRUG | Not Approved |  |
| GDF15 | RESVERATROL | Approved | antineoplastic agent,antidiabetic,for treatment of herpes simplex virus 1 |
| GDF15 | CALCITRIOL | Approved | antineoplastic agent,antipsoriatic agent |
| GDF15 | DICLOFENAC SODIUM | Approved | for treatment of glaucoma,analgesic,NSAID |
| ANGPT2 | TREBANANIB | Not Approved |  |
| ANGPT2 | RIBAVIRIN | Approved |  |
| ANGPT2 | BREMELANOTIDE | Approved | for treatment of sexual dysfunction |
| ANGPT2 | FARICIMAB | Approved |  |
| ANGPT2 | SETMELANOTIDE | Not Approved |  |
| ANGPT2 | CVX-241 | Not Approved |  |
| ANGPT2 | MBP10 | Not Approved |  |
| ANGPT2 | AGOUTI-RELATED PROTEIN | Not Approved |  |
| ANGPT2 | NESVACUMAB | Not Approved |  |
| ANGPT2 | MEDI-3617 | Not Approved |  |
| ANGPT2 | CVX-060 | Not Approved |  |
| ANGPT2 | VANUCIZUMAB | Not Approved |  |
| ANGPT2 | MT-II | Not Approved |  |
| ANGPT2 | [125I]NDP-MSH | Not Approved |  |
| ANGPT2 | CORTICOTROPIN | Approved | Diagnostic Agents |
| ANGPT2 | THIQ | Not Approved |  |
| ANGPT2 | SHU9119 | Not Approved |  |
| ANGPT2 | MCL0129 | Not Approved |  |
| ANGPT2 | &ALPHA;-MSH | Not Approved |  |
| ANGPT2 | HS014 | Not Approved |  |
| ANGPT2 | [125I]SHU9119 | Not Approved |  |
| ANGPT2 | HS024 | Not Approved |  |
| ANGPT2 | RY764 | Not Approved |  |
| ANGPT2 | AFAMELANOTIDE | Approved | dermatological agent |
| ANGPT2 | RECOMBINANT VASCULAR ENDOTHELIAL GROWTH FACTOR | Not Approved |  |
| ANGPT2 | NESVACUMAB | Not Approved |  |
| ANGPT2 | AMG-780 | Not Approved |  |
| ANGPT2 | ZANSECIMAB | Not Approved |  |
| ANGPT2 | NESVACUMAB | Not Approved | antineoplastic agent |
| ANGPT2 | PG-901 | Not Approved |  |

**Supplementary Table S6. DrugBank database for the reproducible proteins.**

| Drug target | Drug Names | Drug Type | Action | CAS Number |
| --- | --- | --- | --- | --- |
| NPPB | Carvedilol | Small Molecule | other | 610309-89-2 |
| NPPB | Oxymetholone | Small Molecule | NA | 434-07-1 |
| ANGPT2 | Faricimab | Biotech | antagonist | 1607793-29-2 |
| ANGPT2 | Vanucizumab | Biotech | regulator | 1448221-05-3 |
| IGFBP1 | Mecasermin | Biotech | carrier | 68562-41-4 |
| IGFBP2 | Mecasermin | Biotech | carrier | 68562-41-4 |
| IGFBP7 | Insulin human | Biotech | inhibitor, binder | 11061-68-0 |
| PLAUR | Urokinase | Biotech | inducer, modulator | 9039-53-6 |
| PLAUR | Tenecteplase | Biotech | NA | 191588-94-0 |
| PLAUR | WX-UK1 | Small Molecule | NA | 255374-84-6 |
| PLAUR | Lanoteplase | Biotech | NA | 171870-23-8 |
| PLAUR | Ruxolitinib | Small Molecule | inhibitor | 1092939-17-7 |
| TAGLN | Artenimol | Small Molecule | ligand | 71939-50-9 |

**Supplementary Table S7. Open Targets for the reproducible proteins.**

| DrugId | DrugName | Type | Mechanism Of Action | ActionType | DiseaseId | DiseaseName | Phase | Status | Source |
| --- | --- | --- | --- | --- | --- | --- | --- | --- | --- |
| CHEMBL4650434 | ENIBARCIMAB | Antibody | ADM stabiliser | Stabiliser | EFO_0003144 | heart failure | 2 | Recruiting | https://clinicaltrials.gov/study/NCT04252937 |
| CHEMBL4650434 | ENIBARCIMAB | Antibody | ADM stabiliser | Stabiliser | EFO_0006834 | septic shock | 2 | Completed | https://clinicaltrials.gov/study/NCT03085758 |
| CHEMBL4650434 | ENIBARCIMAB | Antibody | ADM stabiliser | Stabiliser | MONDO_0100096 | COVID-19 | 2 | Terminated | https://clinicaltrials.gov/study/NCT05156671 |
| CHEMBL4297750 | FARICIMAB | Antibody | Angiopoietin-2 inhibitor | Inhibitor | MONDO_0003005 | macular retinal edema | 4 |  | https://www.ema.europa.eu/en/medicines/human/EPAR/vabysmo |
| CHEMBL4297750 | FARICIMAB | Antibody | Angiopoietin-2 inhibitor | Inhibitor | MONDO_0007935 | cystoid macular edema | 4 |  | https://www.accessdata.fda.gov/drugsatfda_docs/label/2022/761235s000lbl.pdf |
| CHEMBL4297750 | FARICIMAB | Antibody | Angiopoietin-2 inhibitor | Inhibitor | EFO_0009321 | diabetic macular edema | 4 | Recruiting | https://clinicaltrials.gov/study/NCT05224102,https://clinicaltrials.gov/study/NCT05610319 |
| CHEMBL4297750 | FARICIMAB | Antibody | Angiopoietin-2 inhibitor | Inhibitor | EFO_0009321 | diabetic macular edema | 4 | Not yet recruiting | https://clinicaltrials.gov/study/NCT05610488 |
| CHEMBL4297750 | FARICIMAB | Antibody | Angiopoietin-2 inhibitor | Inhibitor | EFO_0010977 | macrovascular complications of diabetes | 4 |  | https://www.ema.europa.eu/en/medicines/human/EPAR/vabysmo |
| CHEMBL4297750 | FARICIMAB | Antibody | Angiopoietin-2 inhibitor | Inhibitor | EFO_0004683 | wet macular degeneration | 4 |  | https://www.ema.europa.eu/en/medicines/human/EPAR/vabysmo,https://www.accessdata.fda.gov/drugsatfda_docs/label/2022/761235s000lbl.pdf |
| CHEMBL4297750 | FARICIMAB | Antibody | Angiopoietin-2 inhibitor | Inhibitor | EFO_0005753 | ocular vascular disease | 4 |  | https://www.whocc.no/atc_ddd_index/?code=S01LA09 |
| CHEMBL2108568 | TREBANANIB | Protein | Angiopoietin-2 inhibitor | Inhibitor | MONDO_0002158 | fallopian tube cancer | 3 | Terminated | https://clinicaltrials.gov/study/NCT01493505 |
| CHEMBL2108568 | TREBANANIB | Protein | Angiopoietin-2 inhibitor | Inhibitor | MONDO_0008170 | ovarian cancer | 3 | Completed | https://clinicaltrials.gov/study/NCT01204749 |
| CHEMBL4297750 | FARICIMAB | Antibody | Angiopoietin-2 inhibitor | Inhibitor | EFO_0001365 | age-related macular degeneration | 3 | Active, not recruiting | https://clinicaltrials.gov/study/NCT04777201 |
| CHEMBL2108568 | TREBANANIB | Protein | Angiopoietin-2 inhibitor | Inhibitor | MONDO_0002087 | peritoneum cancer | 3 | Terminated | https://clinicaltrials.gov/study/NCT01493505 |
| CHEMBL2108568 | TREBANANIB | Protein | Angiopoietin-2 inhibitor | Inhibitor | MONDO_0015686 | primary peritoneal carcinoma | 3 | Completed | https://clinicaltrials.gov/study/NCT01204749 |
| CHEMBL4297750 | FARICIMAB | Antibody | Angiopoietin-2 inhibitor | Inhibitor | EFO_0001365 | age-related macular degeneration | 3 | Recruiting | https://clinicaltrials.gov/study/NCT05904028 |
| CHEMBL4297750 | FARICIMAB | Antibody | Angiopoietin-2 inhibitor | Inhibitor | EFO_0004683 | wet macular degeneration | 3 | Completed | https://clinicaltrials.gov/study/NCT03823300,https://clinicaltrials.gov/study/NCT03823287 |
| CHEMBL2108568 | TREBANANIB | Protein | Angiopoietin-2 inhibitor | Inhibitor | MONDO_0008170 | ovarian cancer | 3 | Terminated | https://clinicaltrials.gov/study/NCT01493505 |
| CHEMBL2108568 | TREBANANIB | Protein | Angiopoietin-2 inhibitor | Inhibitor | MONDO_0002158 | fallopian tube cancer | 3 | Completed | https://clinicaltrials.gov/study/NCT01204749 |
| CHEMBL4297750 | FARICIMAB | Antibody | Angiopoietin-2 inhibitor | Inhibitor | EFO_0009321 | diabetic macular edema | 3 | Completed | https://clinicaltrials.gov/study/NCT03622580,https://clinicaltrials.gov/study/NCT03622593,https://clinicaltrials.gov/study/NCT04432831 |
| CHEMBL2108678 | NESVACUMAB | Antibody | Angiopoietin-2 inhibitor | Inhibitor | EFO_0001365 | age-related macular degeneration | 2 | Completed | https://clinicaltrials.gov/study/NCT02713204 |
| CHEMBL4297750 | FARICIMAB | Antibody | Angiopoietin-2 inhibitor | Inhibitor | EFO_0009321 | diabetic macular edema | 2 | Completed | https://clinicaltrials.gov/study/NCT02699450,https://clinicaltrials.gov/study/NCT04597918 |
| CHEMBL2108568 | TREBANANIB | Protein | Angiopoietin-2 inhibitor | Inhibitor | EFO_0000182 | hepatocellular carcinoma | 2 | Completed | https://clinicaltrials.gov/study/NCT00872014 |
| CHEMBL2108568 | TREBANANIB | Protein | Angiopoietin-2 inhibitor | Inhibitor | EFO_1001465 | gliosarcoma | 2 | Completed | https://clinicaltrials.gov/study/NCT01609790 |
| CHEMBL2108568 | TREBANANIB | Protein | Angiopoietin-2 inhibitor | Inhibitor | EFO_0000681 | renal cell carcinoma | 2 | Completed | https://clinicaltrials.gov/study/NCT01664182,https://clinicaltrials.gov/study/NCT00853372,https://clinicaltrials.gov/study/NCT00467025 |
| CHEMBL2108568 | TREBANANIB | Protein | Angiopoietin-2 inhibitor | Inhibitor | MONDO_0015686 | primary peritoneal carcinoma | 2 | Completed | https://clinicaltrials.gov/study/NCT00479817 |
| CHEMBL2108568 | TREBANANIB | Protein | Angiopoietin-2 inhibitor | Inhibitor | EFO_0003833 | brain neoplasm | 2 | Completed | https://clinicaltrials.gov/study/NCT01609790 |
| CHEMBL2108568 | TREBANANIB | Protein | Angiopoietin-2 inhibitor | Inhibitor | EFO_1001968 | soft tissue sarcoma | 2 | Completed | https://clinicaltrials.gov/study/NCT01623869 |
| CHEMBL2108568 | TREBANANIB | Protein | Angiopoietin-2 inhibitor | Inhibitor | MONDO_0007254 | breast cancer | 2 | Completed | https://clinicaltrials.gov/study/NCT00511459 |
| CHEMBL3545259 | VANUCIZUMAB | Antibody | Angiopoietin-2 inhibitor | Inhibitor | MONDO_0005575 | colorectal cancer | 2 | Terminated | https://clinicaltrials.gov/study/NCT02141295 |
| CHEMBL4594470 | CVX-060 | Unknown | Angiopoietin-2 inhibitor | Inhibitor | EFO_0000681 | renal cell carcinoma | 2 | Terminated | https://clinicaltrials.gov/study/NCT01441414 |
| CHEMBL4297750 | FARICIMAB | Antibody | Angiopoietin-2 inhibitor | Inhibitor | EFO_0001365 | age-related macular degeneration | 2 | Completed | https://clinicaltrials.gov/study/NCT03038880 |
| CHEMBL4650486 | ZANSECIMAB | Antibody | Angiopoietin-2 inhibitor | Inhibitor | EFO_0003106 | pneumonia | 2 | Terminated | https://clinicaltrials.gov/study/NCT04342897 |
| CHEMBL2108568 | TREBANANIB | Protein | Angiopoietin-2 inhibitor | Inhibitor | EFO_0000673 | prostate adenocarcinoma | 2 | Completed | https://clinicaltrials.gov/study/NCT01553188 |
| CHEMBL4594470 | CVX-060 | Unknown | Angiopoietin-2 inhibitor | Inhibitor | EFO_0000519 | glioblastoma multiforme | 2 | Withdrawn | https://clinicaltrials.gov/study/NCT01225510 |
| CHEMBL4297750 | FARICIMAB | Antibody | Angiopoietin-2 inhibitor | Inhibitor | EFO_0009760 | non-proliferative diabetic retinopathy | 2 | Active, not recruiting | https://clinicaltrials.gov/study/NCT05681884 |
| CHEMBL2108568 | TREBANANIB | Protein | Angiopoietin-2 inhibitor | Inhibitor | EFO_0000519 | glioblastoma multiforme | 2 | Completed | https://clinicaltrials.gov/study/NCT01609790 |
| CHEMBL2108568 | TREBANANIB | Protein | Angiopoietin-2 inhibitor | Inhibitor | EFO_0008528 | urothelial carcinoma | 2 | Withdrawn | https://clinicaltrials.gov/study/NCT01907308 |
| CHEMBL2108568 | TREBANANIB | Protein | Angiopoietin-2 inhibitor | Inhibitor | MONDO_0002158 | fallopian tube cancer | 2 | Completed | https://clinicaltrials.gov/study/NCT00479817 |
| CHEMBL4297750 | FARICIMAB | Antibody | Angiopoietin-2 inhibitor | Inhibitor | MONDO_0810000 | choroidal neovascularization | 2 | Completed | https://clinicaltrials.gov/study/NCT02484690 |
| CHEMBL2108678 | NESVACUMAB | Antibody | Angiopoietin-2 inhibitor | Inhibitor | EFO_0009321 | diabetic macular edema | 2 | Completed | https://clinicaltrials.gov/study/NCT02712008 |
| CHEMBL2108568 | TREBANANIB | Protein | Angiopoietin-2 inhibitor | Inhibitor | EFO_0000632 | oligodendroglioma | 2 | Completed | https://clinicaltrials.gov/study/NCT01609790 |
| CHEMBL2108568 | TREBANANIB | Protein | Angiopoietin-2 inhibitor | Inhibitor | MONDO_0008170 | ovarian cancer | 2 | Completed | https://clinicaltrials.gov/study/NCT00479817 |
| CHEMBL2108568 | TREBANANIB | Protein | Angiopoietin-2 inhibitor | Inhibitor | EFO_0003968 | angiosarcoma | 2 | Completed | https://clinicaltrials.gov/study/NCT01623869 |
| CHEMBL2108568 | TREBANANIB | Protein | Angiopoietin-2 inhibitor | Inhibitor | EFO_1000218 | Digestive System Carcinoma | 2 | Completed | https://clinicaltrials.gov/study/NCT00583674 |
| CHEMBL4650486 | ZANSECIMAB | Antibody | Angiopoietin-2 inhibitor | Inhibitor | MONDO_0100096 | COVID-19 | 2 | Terminated | https://clinicaltrials.gov/study/NCT04342897 |
| CHEMBL3545259 | VANUCIZUMAB | Antibody | Angiopoietin-2 inhibitor | Inhibitor | EFO_0000616 | neoplasm | 1 | Completed | https://clinicaltrials.gov/study/NCT02665416,https://clinicaltrials.gov/study/NCT01688206 |
| CHEMBL4594470 | CVX-060 | Unknown | Angiopoietin-2 inhibitor | Inhibitor | EFO_0000313 | carcinoma | 1 | Completed | https://clinicaltrials.gov/study/NCT00879684 |
| CHEMBL2109298 | MEDI-3617 | Antibody | Angiopoietin-2 inhibitor | Inhibitor | EFO_0000616 | neoplasm | 1 | Completed | https://clinicaltrials.gov/study/NCT01248949 |
| CHEMBL2109298 | MEDI-3617 | Antibody | Angiopoietin-2 inhibitor | Inhibitor | EFO_0002617 | metastatic melanoma | 1 | Completed | https://clinicaltrials.gov/study/NCT02141542 |
| CHEMBL2108678 | NESVACUMAB | Antibody | Angiopoietin-2 inhibitor | Inhibitor | EFO_0000616 | neoplasm | 1 | Completed | https://clinicaltrials.gov/study/NCT01271972,https://clinicaltrials.gov/study/NCT01688960 |
| CHEMBL2108568 | TREBANANIB | Protein | Angiopoietin-2 inhibitor | Inhibitor | EFO_0003060 | non-small cell lung carcinoma | 1 | Completed | https://clinicaltrials.gov/study/NCT01666977 |
| CHEMBL4297750 | FARICIMAB | Antibody | Angiopoietin-2 inhibitor | Inhibitor | EFO_0009606 | macular degeneration | 1 | Completed | https://clinicaltrials.gov/study/NCT01941082 |
| CHEMBL2108568 | TREBANANIB | Protein | Angiopoietin-2 inhibitor | Inhibitor | EFO_1000158 | Central Nervous System Neoplasm | 1 | Completed | https://clinicaltrials.gov/study/NCT01538095 |
| CHEMBL4594470 | CVX-060 | Unknown | Angiopoietin-2 inhibitor | Inhibitor | MONDO_0004992 | Cancer | 1 | Completed | https://clinicaltrials.gov/study/NCT00879684 |
| CHEMBL2109586 | AMG-780 | Antibody | Angiopoietin-2 inhibitor | Inhibitor | EFO_0000616 | Neoplasm | 1 | Terminated | https://clinicaltrials.gov/study/NCT01137552 |
| CHEMBL4650486 | ZANSECIMAB | Antibody | Angiopoietin-2 inhibitor | Inhibitor | EFO_0000616 | Neoplasm | 1 | Completed | https://clinicaltrials.gov/study/NCT02597036 |
| CHEMBL2108568 | TREBANANIB | Protein | Angiopoietin-2 inhibitor | Inhibitor | EFO_0000616 | Neoplasm | 1 | Active, not recruiting | https://clinicaltrials.gov/study/NCT03239145 |
| CHEMBL2108568 | TREBANANIB | Protein | Angiopoietin-2 inhibitor | Inhibitor | EFO_0000616 | Neoplasm | 1 | Completed | https://clinicaltrials.gov/study/NCT01992341,https://clinicaltrials.gov/study/NCT02525536,https://clinicaltrials.gov/study/NCT00861419,https://clinicaltrials.gov/study/NCT00102830 |

**Supplementary Table S8. Genetic association of 13** **reproducible proteins on AF and atrial traits.**

| Protein | AF (Consortium) | |  | AF (FinnGen) | |  | BSA indexed LAmax | |  | BSA indexed LAmin | |  | BSA indexed LASV | |  | LAEF | |
| --- | --- | --- | --- | --- | --- | --- | --- | --- | --- | --- | --- | --- | --- | --- | --- | --- | --- |
|  | OR (95%CI) | P value |  | OR (95%CI) | P value |  | OR (95%CI) | P value |  | OR (95%CI) | P value |  | OR (95%CI) | P value |  | OR (95%CI) | P value |
| NT-proBNP | 1.03 (0.98-1.08) | 0.272 |  | 1.07 (1.00-1.14) | 0.044 |  | 0.97 (0.92- 1.02) | 0.18 |  | 0.98 (0.93-1.03) | 0.397 |  | 0.96 (0.91-1.02) | 0.174 |  | 1.00 (0.95-1.05) | 0.874 |
| ANGPT2 | 1.02 (0.93-1.12) | 0.646 |  | 1.06 (0.95-1.20) | 0.288 |  | — | — | — | — | — | — | — | — | — | — | — |
| BNP | 1.09 (0.94-1.26) | 0.264 |  | 1.16 (0.95-1.43) | 0.146 |  | 0.87 (0.74-1.03) | 0.105 |  | 0.94 (0.80-1.11) | 0.457 |  | 0.86 (0.73-1.01) | 0.071 |  | 0.95 (0.81-1.12) | 0.53 |
| ADM | — | — | — | — | — | — | — | — | — | — | — | — | — | — | — | — | — |
| GDF15 | 0.99 (0.95-1.04) | 0.661 |  | 1.03 (0.98-1.08) | 0.214 |  | — | — |  | 1.01 (0.97-1.05) | 0.522 |  | — | — |  | 0.97 (0.94-1.01) | 0.17 |
| IGFBP-1 | 0.94 (0.81-1.09) | 0.413 |  | 1.15 (0.93-1.43) | 0.19 |  | 0.85 (0.72-1.01) | 0.061 |  | 0.87 (0.74-1.03) | 0.104 |  | 0.89 (0.74-1.05) | 0.16 |  | 1.06 (0.90-1.26) | 0.483 |
| IGFBP-2 | 1.11 (0.86-1.44) | 0.419 |  | 0.80 (0.57-1.10) | 0.172 |  | 1.02 (0.79-1.31) | 0.897 |  | 0.99 (0.77-1.28) | 0.943 |  | 1.04 (0.80-1.35) | 0.767 |  | 1.00 (0.80-1.29) | 0.99 |
| IGFBP-7 | — | — |  | — | — |  | 1.00 (0.96-1.05) | 0.859 |  | — | — |  | 1.01 (0.96-1.06) | 0.709 |  | — | — |
| PLAUR | 1.02 (0.96-1.09) | 0.513 |  | — | — |  | 0.97 (0.88-1.07) | 0.579 |  | 1.00 (0.92-1.08) | 0.972 |  | 0.95 (0.86-1.06) | 0.357 |  | — | — |
| RSPO4 | — | — |  | — | — |  | 1.00 (0.88-1.13) | 0.98 |  | 0.96 (0.86-1.08) | 0.506 |  | — | — |  | 1.08 (0.97-1.22) | 0.163 |
| SVEP1 | 1.01 (0.98-1.05) | 0.476 |  | 1.03 (0.99-1.08) | 0.13 |  | — | — |  | 1.01 (0.97-1.05) | 0.63 |  | — | — |  | 0.98 (0.94-1.02) | 0.288 |
| TAGLN | 0.72 (0.55-0.95) | 0.018 |  | 0.96 (0.72-1.28) | 0.769 |  | 1.14 (0.89-1.47) | 0.302 |  | 1.11 (0.78-1.58) | 0.55 |  | 1.15 (0.95-1.39) | 0.155 |  | 0.98 (0.71-1.35) | 0.885 |
| SPON1 | 0.98 (0.89-1.09) | 0.707 |  | — | — |  | 0.96 (0.92-1.01) | 0.115 |  | 0.96 (0.91-1.01) | 0.083 |  | 0.98 (0.93-1.03) | 0.367 |  | 0.97 (0.94-1.01) | 0.17 |

AF, atrial fibrillation; BSA, body surface area; LAmax, maximum left atrial volume; LAmin, minimum left atrial volume; LASV, left atrial stroke volume; LAEF, left atrial emptying fraction; OR, odds ratio; CI, confidence interval.

The missing values marked with “-” represent the genes without effective cis-pQTLs in the MR analysis.

**Supplementary Table S9. Effect of plasma and tissue-specific protein-coding gene expression on AF risk for the 13 reproducible proteins.**

| Gene symbol | Gene ID | Chr | eQTLGen (blood) | | |  | GTEx (Whole Blood) | | |  | GTEx (Left Ventricle) | | |  | GTEx (Atrial Appendage) | | |
| --- | --- | --- | --- | --- | --- | --- | --- | --- | --- | --- | --- | --- | --- | --- | --- | --- | --- |
|  |  |  | Top SNP | OR (95%CI) | P value |  | Top SNP | OR (95%CI) | P value |  | Top SNP | OR (95%CI) | P value |  | Top SNP | OR (95%CI) | P value |
| NPPB | ENSG00000120937 | 1 | — | — | — |  | — | — | — |  | — | — | — |  | — | — | — |
| ANGPT2 | ENSG00000091879 | 8 | rs11990038 | 1.08 (0.91-1.27) | 0.394 |  | — | — | — |  | — | — | — |  | — | — | — |
| ADM | ENSG00000148926 | 11 | rs4910108 | 1.03 (0.99-1.08) | 0.114 |  | — | — | — |  | — | — | — |  | — | — | — |
| GDF15 | ENSG00000130513 | 19 | rs4808795 | 0.97 (0.90-1.04) | 0.424 |  | rs7226 | 0.99 (0.94-1.03) | 0.561 |  | — | — | — |  | — | — | — |
| IGFBP1 | ENSG00000146678 | 7 | — | — | — |  | — | — | — |  | — | — | — |  | — | — | — |
| IGFBP2 | ENSG00000115457 | 2 | rs17496232 | 1.00 (0.96-1.04) | 0.946 |  | rs17496232 | 1.00 (0.98-1.02) | 0.946 |  | — | — | — |  | — | — | — |
| IGFBP7 | ENSG00000163453 | 4 | rs2271808 | 0.87 (0.81-0.93) | 3.14E-05 |  | — | — | — |  | rs35551730 | 0.97 (0.92-1.03) | 0.313 |  | — | — | — |
| PLAUR | ENSG00000011422 | 19 | rs10402946 | 0.94 (0.80-1.11) | 0.47 |  | — | — | — |  |  |  |  |  | — | — | — |
| RSPO4 | ENSG00000101282 | 20 | rs6039500 | 0.97 (0.88-1.07) | 0.556 |  | rs6039500 | 0.99 (0.95-1.03) | 0.556 |  | rs6056616 | 1.00 (1.97-1.03) | 0.864 |  | — | — | — |
| SVEP1 | ENSG00000165124 | 9 | — | — | — |  | — | — | — |  | — | — | — |  | — | — | — |
| TAGLN | ENSG00000149591 | 11 | rs734104 | 0.97 (0.95-1.00) | 0.058 |  | rs6589571 | 0.97 (0.94-1.00) | 0.062 |  | — | — | — |  | — | — | — |
| SPON1 | ENSG00000152268 | 11 | — | — | — |  | — | — | — |  | — | — | — |  | — | — | — |

AF, atrial fibrillation; SNP, single nucleotide polymorphism; OR, odds ratio; CI, confidence interval.

The missing values marked with “-” represent the genes without effective eQTLs in the SMR analysis.

**Supplementary Table S10. Results of colocalization of the 13 reproducible proteins with AF.**

| Gene | nsnps | PP.H0.abf | PP.H1.abf | PP.H2.abf | PP.H3.abf | PP.H4.abf |
| --- | --- | --- | --- | --- | --- | --- |
| ANGPT2 | 1432 | 4.31E-13 | 0.9235768 | 1.66E-14 | 0.0354585 | 0.0409647 |
| ADM | 3461 | 1.08E-301 | 0.8418326 | 1.61E-302 | 0.1253122 | 0.0328552 |
| GDF15 | 1223 | 5.91E-89 | 0.3879606 | 9.25E-89 | 0.6071647 | 0.0048748 |
| IGFBP2 | 1380 | 5.02E-272 | 0.7648088 | 1.49E-272 | 0.2267871 | 0.0084041 |
| IGFBP7 | 2432 | 6.66E-136 | 0.014276 | 6.90E-135 | 0.1469077 | 0.8388164 |
| PLAUR | 1936 | 2.40E-24 | 0.9445624 | 1.14E-25 | 0.0449608 | 0.0104768 |
| RSPO4 | 805 | 1.62E-82 | 0.9551332 | 5.23E-84 | 0.0308071 | 0.0140597 |
| TAGLN | 4034 | 1.53E-300 | 0.2763371 | 2.43E-300 | 0.43859 | 0.2850729 |
| SPON1 | 2833 | 9.78E-271 | 0.0117615 | 8.21E-269 | 0.9881203 | 0.0001182 |


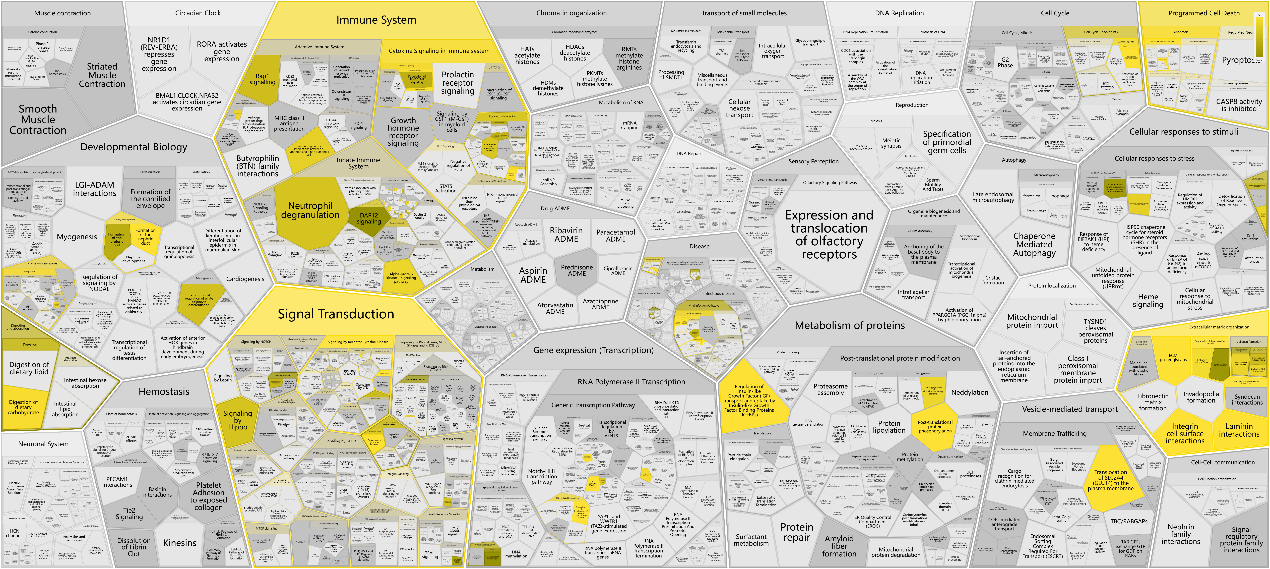


Supplementary Figure S1. Enrichment analysis of all AF-related proteins based on Reactome.


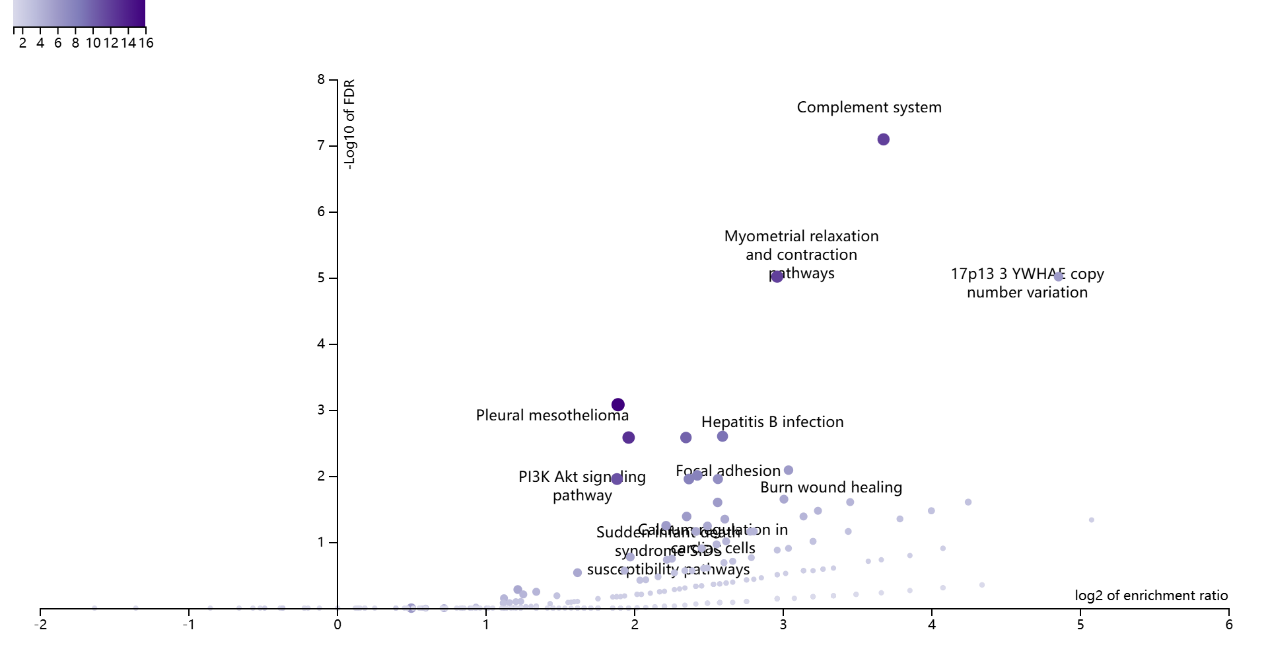


Supplementary Figure S2. Enrichment analysis of all AF-related proteins based on WikiPathways.
